# Exploring functional connectivity in large-scale brain networks in obsessive-compulsive disorder: a systematic review of EEG and fMRI studies

**DOI:** 10.1093/cercor/bhae327

**Published:** 2024-08-16

**Authors:** M Prabhavi N Perera, Efstathia S Gotsis, Neil W Bailey, Bernadette M Fitzgibbon, Paul B Fitzgerald

**Affiliations:** College of Health and Medicine, Australian National University, Building 4, The Canberra Hospital, Hospital Rd, Garran ACT 2605, Australia; Monarch Research Institute, Monarch Mental Health Group, Level 4, 131 York Street Sydney NSW 2000, Australia; College of Health and Medicine, Australian National University, Building 4, The Canberra Hospital, Hospital Rd, Garran ACT 2605, Australia; Monarch Research Institute, Monarch Mental Health Group, Level 4, 131 York Street Sydney NSW 2000, Australia; College of Health and Medicine, Australian National University, Building 4, The Canberra Hospital, Hospital Rd, Garran ACT 2605, Australia; Monarch Research Institute, Monarch Mental Health Group, Level 4, 131 York Street Sydney NSW 2000, Australia; College of Health and Medicine, Australian National University, Building 4, The Canberra Hospital, Hospital Rd, Garran ACT 2605, Australia; Monarch Research Institute, Monarch Mental Health Group, Level 4, 131 York Street Sydney NSW 2000, Australia; College of Health and Medicine, Australian National University, Building 4, The Canberra Hospital, Hospital Rd, Garran ACT 2605, Australia; Monarch Research Institute, Monarch Mental Health Group, Level 4, 131 York Street Sydney NSW 2000, Australia

**Keywords:** obsessive-compulsive disorder, functional connectivity, electroencephalography, functional magnetic resonance imaging, default mode network

## Abstract

Obsessive-compulsive disorder (OCD) is a debilitating psychiatric condition that is difficult to treat due to our limited understanding of its pathophysiology. Functional connectivity in brain networks, as evaluated through neuroimaging studies, plays a pivotal role in understanding OCD. While both electroencephalography (EEG) and functional magnetic resonance imaging (fMRI) have been extensively employed in OCD research, few have fully synthesized their findings. To bridge this gap, we reviewed 166 studies (10 EEG, 156 fMRI) published up to December 2023. In EEG studies, OCD exhibited lower connectivity in delta and alpha bands, with inconsistent findings in other frequency bands. Resting-state fMRI studies reported conflicting connectivity patterns within the default mode network (DMN) and sensorimotor cortico-striato-thalamo-cortical (CSTC) circuitry. Many studies observed decreased resting-state connectivity between the DMN and salience network (SN), implicating the 'triple network model' in OCD. Task-related hyperconnectivity within the DMN-SN and hypoconnectivity between the SN and frontoparietal network suggest OCD-related cognitive inflexibility, potentially due to triple network dysfunction. In conclusion, our review highlights diverse connectivity differences in OCD, revealing complex brain network interplay that contributes to symptom manifestation. However, the presence of conflicting findings underscores the necessity for targeted research to achieve a comprehensive understanding of the pathophysiology of OCD.

## Introduction

Obsessive-compulsive disorder (OCD), affecting approximately 2–3% of the global population (Kessler et al. 2012), presents a complex mental health challenge characterized by the presence of distressing obsessions and compulsions (Stein et al. 2019). OCD exerts a profound impact on various aspects of an individual’s life, with compulsions often demanding a significant time commitment, disrupting daily activities and relationships, thereby compromising overall quality of life (Remmerswaal et al. 2016). The total annual economic burden of OCD was estimated to be US$8.4 billion in the United States, accounting for 5.7% of the cost of treating psychiatric conditions (DuPont et al. 1995). Furthermore, Swedish national survey data show that 11% of OCD patients have been unemployed for over 180 days each year (Pérez-Vigil et al. 2019).

Accepted treatment approaches for OCD primarily involve a combination of pharmacotherapy and psychotherapy. While these traditional treatments have proven effective for many individuals, a significant portion (up to 60%) may not respond adequately (Taylor et al. 2012). This treatment resistance has prompted exploration into alternative approaches, such as transcranial magnetic stimulation (TMS) (Perera et al. 2021), transcranial alternating current stimulation (tACS) (Perera et al. 2023a) and deep brain stimulation (DBS) (Alonso et al. 2015). These therapies are believed to operate by modulating the functional connections within the brain.

Although substantial research has been conducted, the underlying pathophysiological mechanisms of OCD are still not fully understood, which has hindered development of effective treatments. Therefore, there is a critical unmet need to uncover the pathophysiological basis of OCD, which will ultimately pave the way for more effective targeted treatments. The brain is thought to process information, control functions and generate thoughts, emotions and behaviors through interactions within and between intricate networks (Bullmore and Sporns 2009). Prior research extensively investigates the hypothesis that OCD stems from dysfunctional neurocircuitry, marked by abnormal interactions within and between various brain structures. Neuroimaging techniques such as electroencephalography (EEG) and functional magnetic resonance imaging (fMRI) have shown electrophysiological and structural differences in OCD (Menzies et al. 2008; Perera et al. 2019). These findings encompass differences in EEG-measured oscillations (Perera et al. 2023b), event-related potentials (Perera et al. 2023c), and fMRI-measured brain volumes and blood flow activations (Menzies et al. 2008). However, the examination of temporal correlations and interactions between brain regions necessitates the use of functional connectivity (FC) measures, typically conducted through techniques such as EEG or, more commonly, fMRI. FC refers to statistical dependencies or correlations between different brain regions or neural populations and is a measure of the synchronized activity between these regions (Craddock et al. 2015).

Alterations in FC within and between brain networks in OCD have been reported in many studies. Several brain networks have been consistently reported as dysfunctional in OCD. One prominent example is the default mode network (DMN), a large-scale brain network that is active during wakeful rest or while processing detailed thoughts related to external task performance (Raichle 2015). The cortico-striato-thalamo-cortical (CSTC) circuitry includes multiple sub-circuits (e.g. limbic, sensorimotor, dorsal attention loops) which play pivotal roles in a range of brain functions (Graybiel and Rauch 2000). The salience network (SN), plays a crucial role in directing attention and prioritizing stimuli based on their emotional or sensory significance (Seeley 2019). Finally, the frontoparietal network (FPN), is comprised of a complex group of brain regions related to attention, working memory and cognitive control (Marek and Dosenbach 2022). A large body of research reports that dysconnectivity within CSTC circuits is involved in the pathophysiology of OCD (Pauls et al. 2014). However, aberrant FC between the DMN, SN and FPN has also been linked to a “triple network model” (TNM) of psychopathology in OCD (Fan et al. 2017a). In this model, the SN acts as a mediator between the FPN and the DMN, which are anticorrelated to each other (when one network is active, the other is suppressed).

A meta-analysis of resting-state network FC in OCD reported dysconnectivity between the DMN, SN and FPN, providing evidence for the TNM dysfunction model in OCD (Gürsel et al. 2018). Another recent meta-analysis has provided supporting evidence towards the traditional dysfunctional CSTC with findings of dysconnectivity within the fronto-limbic CSTC subcircuit (Liu et al. 2022). Furthermore, a systematic review of 20 resting-state fMRI studies highlighted the role of impaired DMN and FPN connectivity in OCD (Fornaro and Vallesi 2023). However, these reviews have not collectively assessed all available studies, and all omitted task-related fMRI findings from their analyses. Task-related fMRI findings offer valuable insights into dynamic changes in brain activity during specific cognitive tasks, enhancing our understanding of neural mechanisms in OCD. Furthermore, a comprehensive review of EEG connectivity findings in OCD has not been performed to date. The current systematic review aims to fill this gap by comprehensively collating EEG and fMRI data, and critically evaluating FC differences in OCD compared to healthy controls (HC). Additionally, we discuss the limitations of EEG and fMRI individually, and explore the potential benefits of integrating the two modalities to acquire simultaneous EEG-fMRI data in future studies. This novel approach aims to enhance our ability to comprehend the underlying neurophysiology of OCD. Furthermore, the categorization of studies based on large-scale brain networks and analysis of the consistency of altered FC findings will collectively enhance our understanding of the neurophysiological patterns associated with OCD. This approach will provide a more nuanced and comprehensive perspective, ultimately suggesting potential brain targets for future treatments.

## Materials and methods

### Search strategy

A search of the relevant literature was performed adhering to the Preferred Reporting Items for Systematic Reviews and Meta-Analyses (PRISMA) guidelines (McInnes et al. 2018). A primary search was performed using several electronic databases including MEDLINE, PubMed, Scopus, PsycINFO and Web of Science. Studies published in English up to December 2023 were included. The keywords used for the initial search were obsessive-compulsive disorder, OCD, electroencephalography, EEG, functional magnetic resonance imaging, fMRI, functional connectivity, FC and brain networks. After identifying key networks implicated in OCD through an initial literature search, search terms were expanded to include additional keywords such as default mode network, DMN, salience network, SN, frontoparietal network, FPN, cortico-striato-thalamo-cortical circuit and CSTC. The obtained results were imported into Covidence (https://www.covidence.org/) to facilitate abstract screening, full-text review, and study extraction. A second reviewer (ESG) was involved in the study screening process. Records for which reviewers had opposing decisions were reviewed together and a consensus was formed.

### Study selection

**Fig. 1:**
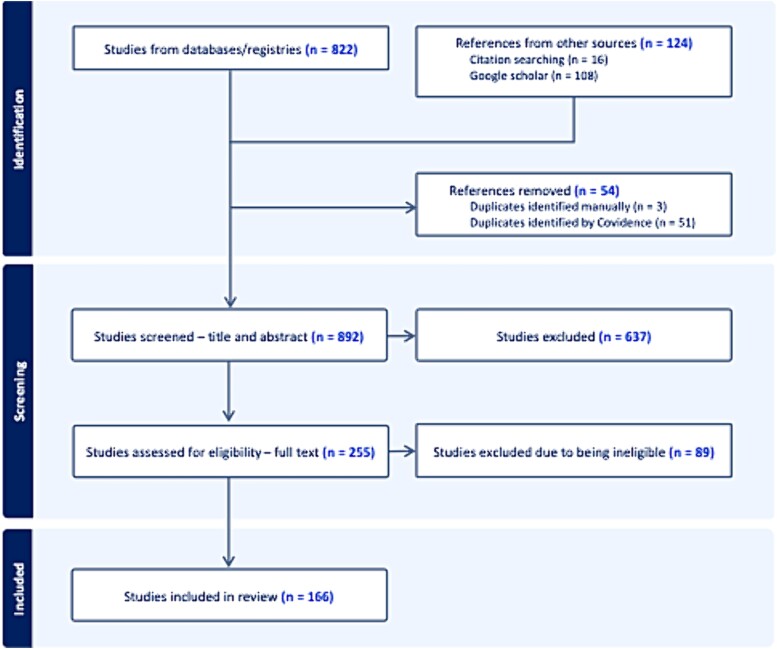
PRISMA diagram of the study selection process.

The inclusion criteria were: (1) availability of full-length articles published in peer-reviewed journals; (2) inclusion of participants with a primary diagnosis of OCD or obsessive-compulsive personality disorder; (3) availability of a detailed description of EEG/fMRI methods and results; (4) the study involved analysis of FC in OCD participants with a comparison sample of HC (note that FC excludes structural MRI examinations of connectivity). Studies conducted with both pediatric and adult populations were included. Initially, all identified articles were included following inspection of the title and the abstract. Thereafter, full-text versions were assessed in more detail to exclude ineligible articles. Additional relevant articles were identified through manual searching of the reference lists of the selected articles. The study inclusion and exclusion procedures are summarized in Fig. 1.

### Data extraction

The following information was extracted and recorded from the included studies: (1) year of publication; (2) EEG/fMRI technique (resting-state or task-related); (3) key findings. The following characteristics of participants in each study were recorded: (a) sample size (experimental and control groups); (b) mean age; (c) male:female ratio. The studies were initially grouped based on the neuroimaging technique (EEG or fMRI). fMRI studies were further grouped based on resting-state/task and specific large-scale brain networks based on their findings. The key findings of each study within each group were extracted to facilitate qualitative analysis, aiming to elucidate the pathophysiology of OCD. In cases where studies did not report specific brain networks involved, the reported brain regions were mapped to commonly accepted functional networks based on widely used atlases such as Yeo’s 7- and 17-network parcellations (Schaefer et al. 2018; Yeo et al. 2011). The brain regions associated with each identified network are highlighted in Supplementary Table S1. The principal FC analysis technique used in each fMRI study is shown in Supplementary Tables S4 and S5.

### Quantitative analysis

A quantitative assessment was conducted exclusively for resting-state fMRI studies to evaluate the consistency of reporting for each finding. Findings indicating higher or lower FC within and between brain networks compared to HC were identified and compiled. Subsequently, the total number of participants with OCD included in each study was summed to derive a final cumulative count for each identified connectivity finding. These cumulative sums were used to generate chord diagrams representing the number of OCD patients that showed the relevant inter/intra-network connectivity finding (Krzywinski et al. 2009). This analysis was not extended to EEG and task-related fMRI studies due to the limited number of available studies and the heterogeneity between studies, respectively.

## Results

The final eligible list included 166 studies (10 EEG studies and 156 fMRI studies). The fMRI studies were further categorized into resting-state (125 studies) and task-related (31 studies) findings.

### E‌EG studies

Table 1 summarizes the OCD and HC participant data and key findings of the EEG studies. All studies reported EEG findings while participants were at rest. Two studies reported significantly lower FC within the delta frequency band (Perera et al. 2023b; Özçoban et al. 2018). Perera et al. (2023b) found reduced delta FC in frontocentral and centroparietal electrodes, while Özçoban et al. (2018) reported decreased delta FC in frontal scalp regions. In contrast, one study reported increased theta FC in the fronto-occipital regions compared to HC (Desarkar et al. 2007). FC within the alpha band was found to be decreased consistently (Choi et al. 2021; Tan et al. 2019; Tan et al. 2022; Velikova et al. 2010). Choi et al. (2021) noted reduced FC within DMN nodes, while Velikova et al. (2010) and Tan et al. (2022) observed decreased inter-hemispheric alpha FC in frontal, central, and temporal electrodes. Beta band FC exhibited conflicting findings across studies, with two reporting decreased FC (Choi et al. 2021; Olbrich et al. 2013) and one indicating an increase in beta FC within bilateral posterior regions (Tan et al. 2019). The discrepancy extends to the localisation of decreased beta FC, with one study identifying it within the DMN (Choi et al. 2021), while another pinpointed it specifically within frontal areas, excluding the DMN (Olbrich et al. 2013). Three studies (Saifutdinova et al. 2016; Tan et al. 2022; Özçoban et al. 2018) reported overall decreased FC globally in OCD groups compared to HC.

**Table 1:** EEG connectivity studies in OCD.

**Author (year)**	**OCD sample (Male, Female, Mean age, SD)**	**Comparison sample (Male, Female, Mean age, SD)**	**EEG methodology**	**Key findings**
(Perera et al. 2023b)	25 (12 M, 13F; 36.24 ± 13.06y)	27 (9 M, 18F; 31.22 ± 10.66y)	EEG recorded while resting with eyes closed for 3 min.	Significantly lower **delta** (0.5-4 Hz) band FC in OCD in bilateral frontocentral and frontal brain regions.
(Özçoban et al. 2018)	17 (8 M, 9F; 29.35 ± 6.95y)	17	EEG recorded while resting with eyes closed for 3 min.	Global field synchronization was significantly lower in **delta** (1.5-6 Hz) band and **full band** (0.7-70 Hz) in OCD, suggesting synchronization disconnection in large-scale brain regions.
(Desarkar et al. 2007)	20 (10 M, 10F; 31.45 ± 8.91y)	19 (10 M, 9F; 29.85 ± 7.78y)	EEG recorded while resting with eyes closed for 20 min.	Significantly higher fronto-occipital **theta** (4–7.5 Hz) coherence in the theta band in OCD compared to HC, indicating hyperactivity at subcortical circuitry.
(Choi et al. 2021)	29 (15 M, 14F; 27.76 ± 9.51y)	29 (14 M, 15F; 30.55 ± 9.7y)	EEG recorded while resting with eyes closed for 4 min.	Significantly low clustering coefficients in the **low-alpha** (8-10 Hz) band within the DMN in OCD. In Phase locking values (PLV), lowered **low-beta** (12-18 Hz) band FC within the DMN.
(Velikova et al. 2010)	37 (15 M, 22F; 31 ± 10.5y)	37 (15 M, 22F; 31.5 ± 10.5y)	EEG recorded while resting with eyes closed for 5 min.	Reduced inter-hemispheric **alpha** (8-12 Hz) coherence in frontal, central and temporal regions.
(Tan et al. 2022)	17 (7 M, 10F; 29.31 ± 4.26y)	17 (8 M, 9F; 29.13 ± 6.71y)	EEG recorded while resting with eyes open and closed (3 min. For each condition)	Whole-brain global synchronization FC decreased in OCD with inhibited intra- and inter-hemispheric FC within the **alpha** (8-12 Hz) band at rest. Decreased FC showed hemispheric asymmetry.
(Tan et al. 2019)	17 (7 M, 10F; 29.31 ± 4.26y)	17 (8 M, 9F; 29.13 ± 6.71y)	EEG recorded while resting with eyes open and closed (3 min. For each condition)	Long range **alpha** (8-12 Hz) FC significantly reduced in bilateral parietal-occipital areas in OCD. **Beta** (13-30 Hz) FC significantly increased only in the eyes open state.
(Olbrich et al. 2013)	30 (13 M, 17F; 34.6 ± 11.9y)	30 (13 M, 17F; 34,5 ± 11.1y)	EEG recorded while resting with eyes closed for 15 min.	Lagged non-linear coherence significantly decreased for **beta2** (20.5-30 Hz) frequency between frontal areas, but not within DMN (Between rSFG and ACC, rSFG and lMFG, rSFG and lSFG).
(Yazdi-Ravandi et al. 2018)	39 (14 M, 25F; 34.76 ± 10.35y)	19 (8 M, 11F; 31.94 ± 8.22y)	EEG recorded while resting with eyes closed	Frontal and temporal/occipital connectivity within the **high beta** (25-30 Hz) band significantly reduced in OCD using the wPLI measure.
(Saifutdinova et al. 2016)	95 (38 M, 57F; 30 ± 5.6y)	96 (54 M, 42F; 29.2 ± 5y)	EEG recorded while resting with eyes closed	Significantly lower clustering coefficient and higher path length in OCD compared to HC, indicating loss of local integration and multi-scale connection, respectively.

*Note.* OCD—obsessive-compulsive disorder, EEG—electroencephalography, M—male, F—female, y—years old, SD—standard deviation, HC—healthy controls, DMN—default mode network, r—right, l—left, SFG—superior frontal gyrus, ACC—anterior cingulate cortex, MFG—medial frontal gyrus, wPLI—weighted phase lag index, FC—functional connectivity.

**Table 2:** Resting-state fMRI connectivity studies in OCD.

**Author (year)**	**OCD sample (Male, Female, Mean age, SD)**	**Comparison sample (Male, Female, Mean age, SD)**	**Key findings**	**Involved neurocircuitry and direction of connectivity findings**
(Abe et al. 2015)	37 (15 M, 22F; 30.4 ± 7.5y)	38 (18 M, 20F; 32.7 ± 9.7y)	Increased FC between OFC and ventral striatum (NAc) in OCD.	CSTC (VMC) $\uparrow$
(Anticevic et al. 2014)	27 (15 M, 12F; 36.37 ± 13.6y)	66 (41 M, 25F; 33 ± 10.4y)	Clusters of decreased FC within L-lateral PFC. Increased FC between R-putamen and L-cerebellum, NAc and ACC.	CSTC (limbic) $\downarrow$ CSTC (SM)—cerebellum $\uparrow$ CSTC (VMC)—SN $\uparrow$
(Apergis-Schoute et al. 2018)	38 (21 M, 17F; 40.7 ± 13.1y)	34 (18 M, 16F; 38.3 ± 13.3y)	Increased FC from vmPFC to temporal and occipital lobes, cerebellum and the motor cortex	CSTC (limbic) $\uparrow$ CSTC (limbic)—cerebellum $\uparrow$
(Armstrong et al. 2016)	21 (13 M, 8F; 12.7 ± 2.8y)	20 (11 M, 9F; 13.4 ± 1.8y)	Less efficient global network connectivity in OCD. Higher internal FC in sensorimotor, supplementary motor and frontal polar cortex.	CSTC (SM) $\uparrow$
(Becker et al. 2023)	23 (0 M, 23F; 14 ± 3.84y)	44 (0 M, 44F; 14.55 ± 3.88y)	Significantly higher FC within the SN and between the SN and CSTC-VMC in OCD.	SN $\uparrow$ SN—CSTC (VMC) $\uparrow$
(Bernstein et al. 2016)	15 (8 M, 7F; 15.3 ± 2.1y)	13 (7 M, 6F; 16 ± 1.8y)	Decreased FC between L-putamen and OFC, IFG, insula and operculum in OCD.	CSTC (SM)—CSTC (VMC) $\downarrow$ CSTC (SM)—SN $\downarrow$
(Beucke et al. 2013)	23 (11 M, 12F; 29.1 ± 9.1y)	23 (11 M, 12F; 28.7 ± 8.9y)	Increased FC between the OFC and subthalamic nucleus, putamen in OCD.	CSTC (VMC) $\uparrow$ CSTC (VMC)—CSTC (SM) $\uparrow$
(Beucke et al. 2014)	46 (20 M, 26F; 30.7 ± 9.4y)	46 (20 M, 26F; 30.3 ± 8.8y)	Significantly reduced connectivity within the dorsal medial prefrontal cortex subsystem of DMN (PCC, dmPFC).	DMN $\downarrow$
(Calzà et al. 2019)	44 (14 M, 30F; 33.32 ± 11.4y)	40 (19 M, 21F; 34.12 ± 8.8y)	Increased FC between several basal ganglia (Subthalamic nucleus, globus pallidus) in OCD.	CSTC (VMC) $\uparrow$ CSTC (limbic) $\uparrow$
(Cano et al. 2018)	86 (43 M, 43F; 34.38 ± 9.4y)	104 (59 M, 45F; 34.18 ± 10.4y)	Increased FC between STN and pre-motor cortex, decreased FC between STN and lenticular nuclei in OCD.	CSTC (SM) $\uparrow$ CSTC (SM)—CSTC (limbic) $\downarrow$
(Cao et al. 2022a)	88 (56 M, 32F; 27.41 ± 6.6y)	88 (56 M, 32F; 25.95 ± 7.8y)	Early onset group: increased amygdala-precuneus and decreased amygdala-OFC FC. Late onset: increased amygdala-PCG.	CSTC (limbic)—FPN $\uparrow$ CSTC (limbic)—CSTC (VMC) $\downarrow$
(Cao et al. 2022b)	92 (57 M, 35F; 29.42 ± 8.7y)	90 (55 M, 35F; 28.34 ± 10.9y)	Decreased FC between amygdala and L-insula. Increased FC between amygdala and SMA, PCG, and superior temporal gyrus.	CSTC (limbic)—SN $\downarrow$ CSTC (limbic)—CSTC (SM) $\uparrow$ CSTC (limbic)—DMN $\uparrow$
(Chen et al. 2016a)	30 (24 M, 6F; 26.23 ± 5.7y)	30 (23 M, 7F; 28.17 ± 7.7y)	Decreased FC within the dorsal cognitive CSTC (caudate, thalamus) and increased FC between caudate and SMA, PCG	CSTC (DC) $\downarrow$ CSTC (DC)—CSTC (SM) $\uparrow$
(Chen et al. 2016b)	30 (24 M, 6F; 26.23 ± 5.7y)	30 (23 M, 7F; 28.17 ± 7.7y)	Increased FC within the FPN in OCD compared to HC.	FPN $\uparrow$
(Chen et al. 2018)	40 (27 M, 13F; 27.28 ± 8.2y)	40 (27 M, 13F; 27 ± 8.3y)	Decreased intrinsic connectivity within SN, and decreased inter-network connectivity between SN and DMN, and FPN.	SN $\downarrow$ SN—DMN $\downarrow$ SN—FPN $\downarrow$
(Chen et al. 2019)	23 (15 M, 8F; 32.1 ± 10.5y)	23 (15 M, 8F; 31.4 ± 10y)	Increased FC from thalamus to dACC, L-SMA and decreased FC to R-middle occipital gyrus.	CSTC (SM) $\uparrow$ CSTC (SM)—SN $\uparrow$ CSTC (SM)—VN $\downarrow$
(Chen et al. 2021)	40 (27 M, 13F; 27.28 ± 8.2y)	38 (25 M, 13F; 27.18 ± 8.3y)	Decreased FC between NAc and BL-OFC, mPFC in OCD.	CSTC (VMC) $\downarrow$ CSTC (VMC)—DMN $\downarrow$
(Cheng et al. 2013)	23 (8 M, 15F; 31 ± 10.26y)	23 (8 M, 15F; 31.65 ± 8.9y)	Increased FC between ACC and SFG, midbrain and SMA, and between PCC and OFG, DLPFC in OCD	SN – CSTC (SM) $\uparrow$ DMN—CSTC (DC) $\uparrow$ DMN—FPN $\uparrow$
(Coutinho et al. 2016)	10 (5 M, 5F; 40 ± 9.4y)	10 (5 M, 5F; 38 ± 8.8y)	Increased FC within the DMN (PCC, precuneus, mPFC, BL-inferior parietal cortex) in OCPD compared to HC	DMN $\uparrow$
(Cui et al. 2020)	40 (27 M, 13F; 27.28 ± 8.2y)	38 (25 M, 13F; 27.18 ± 8.3y)	Decreased FC within the DMN (L-PCC/lingual gyrus) and SM network (PCG) and increased FC within the FPN (DLPFC).	DMN $\downarrow$ CSTC (SM) $\downarrow$ FPN $\uparrow$
(Cyr et al. 2020)	25 (12 M, 13F; 12.8 ± 2.9y)	23 (12 M, 11F; 11 ± 3.3y)	Decreased FC between L-angular gyrus and middle frontal gyrus in OCD.	DMN – FPN $\downarrow$
(Cyr et al. 2021)	25 (12 M, 13F; 12.8 ± 2.9y)	23 (11 M, 12F; 11 ± 3.3y)	Decreased FC between R-amygdala and vmPFC in OCD.	CSTC (limbic) $\downarrow$
(de Vries et al. 2019)	39 (18 M, 21F; 38 ± 9.7y)	36 (17 M, 19F; 39.4 ± 11.3y)	Significantly higher FC within the fronto-limbic network (vmPFC to basal ganglia) in OCD.	CSTC (limbic) $\uparrow$
(Deng et al. 2019)	46 (26 M, 20F; 30.39 ± 10.7y)	46 (26 M, 20F; 31.83 ± 10.3y)	Decreased FC in lingual gyrus, PCG, putamen in OCD.	CSTC (SM) $\downarrow$ CSTC (SM) – DMN $\downarrow$
(Dikmeer et al. 2021)	30 (13 M, 17F; 32.4 ± 10y)	31 (13 M, 18F; 32.3 ± 8.5y)	Significantly reduced FC between caudate and middle temporal/middle occipital cortex.	CSTC (DC) – VN $\downarrow$ CSTC (DC)—DMN $\downarrow$
(Ding et al. 2023)	50 (29 M, 21F; 26.36 ± 8y)	50 (32 M, 18F; 25.60 ± 7.9y)	Decreased dynamic FC between L-superior temporal gyrus and cerebellum, and between R-SMA and R-DLPFC.	DMN – Cerebellum $\downarrow$ CSTC (SM)—FPN $\downarrow$
(Dong et al. 2020)	35 (24 M, 11F; 23.6 ± 5.5y)	35 (18 M, 17F; 27.8 ± 6.7y)	Increased FC between L-caudate and OFC in OCD.	CSTC (DC) – CSTC (VMC) $\uparrow$
(Fajnerova et al. 2020)	36 (18 M, 18F; 33.26 ± 8.2y)	36 (19 M, 17F; 33.26 ± 6.7y)	Increased FC between precuneus-angular gyrus and DLPFC. Decreased FC between caudate-thalamus and ACC-limbic lobe.	CSTC (DC) $\downarrow$ FPN – DMN $\uparrow$ SN—CSTC (limbic) $\downarrow$
(Fan et al. 2017b)	40 (26 M, 14F; 22.89 ± 5.6y)	24 (9 M, 15F; 21.92 ± 2.2y)	Significantly increased FC within the SN (R-anterior insula, L-dACC) in OCD group with good insight. Decreased connectivity between R-AI and mOFC in poor insight group.	SN $\uparrow$ SN – CSTC (VMC) $\downarrow$
(Fan et al. 2017a)	35 (19 M, 16F; 24.23 ± 5.6y)	32 (12 M, 20F; 22.53 ± 2.2y)	Increased FC within the DMN, FPN, SN and between SN-DMN and SN-FPN in OCD.	DMN $\uparrow$FPN $\uparrow$ SN $\uparrow$ SN – DMN $\uparrow$ SN—FPN $\uparrow$
(Fan et al. 2018)	35 (19 M, 16F; 23.86 ± 5.5y)	36 (13 M, 23F; 22.86 ± 2.7y)	Significantly reduced FC between R-mPFC with SFG and BL-thalamus in OCD	DMN – FPN $\downarrow$
(Fan et al. 2023)	165 (84 M, 81F; 23.65 ± 6.7y)	79 (35 M, 44F; 23.78 ± 5.4y)	Decreased FC within the response inhibition network involving medial prefrontal cortex and inferior parietal lobe.	DMN $\downarrow$
(Fitzgerald et al. 2011)	60 (27 M, 33F; 19.75 ± 3.03y)	61 (28 M, 33F; 19.83 ± 3.48y)	Increased FC in dorsal cognitive CSTC (dorsal striatum, vmPFC) in all age groups. Youngest age group showed decreased FC in CSTC loops involved in cognitive control (dorsal striatum/thalamus, dACC).	CSTC (DC) $\uparrow$ CSTC (DC) – DMN $\downarrow$
(Fullana et al. 2017)	73 (35 M, 38F; 34.18 ± 9.3y)	84 (41 M, 43F; 33.68 ± 9.8y)	Decreased FC between basolateral amygdala and vmPFC in OCD.	CSTC (limbic) $\downarrow$
(Gao et al. 2019)	64 (36 M, 28F; 29 ± 6.9y)	60 (31 M, 29F; 28.5 ± 5.4y)	Significantly increased FC between the L-DLPFC and L-cerebellum in OCD.	CSTC (DC) – cerebellum $\uparrow$
(Gao et al. 2021)	45 (25 M, 20F; 28.7 ± 6.7y)	40 (22 M, 18F; 28.9 ± 6.4y)	Increased FC between L-amygdala and R-middle frontal gyrus, amygdala and R-cuneus.	CSTC (limbic) – FPN $\uparrow$ CSTC (limbic)—VN $\uparrow$
(Geffen et al. 2022)	24 (13 M, 11F; 37.2 ± 11.9y)	33 (15 M, 18F; 35.7 ± 11.5y)	Decreased FC between SN and DMN, between visual network and both DMN and SN in OCD.	SN – DMN $\downarrow$ VN—DMN $\downarrow$ VN—SN $\downarrow$
(Göttlich et al. 2014)	17 (5 M, 12F; 30.4 ± 9.6y)	19 (4 M, 15F; 32.6 ± 11.6y)	1. Decreased connectivity between the limbic CSTC to DMN, executive/attention network and basal ganglia in OCD. 2. Intra-network connectivity within the limbic network was decreased in OCD 3. Hyperconnectivity within FPN in OCD.	CSTC (limbic) – DMN $\downarrow$ CSTC (limbic)—FPN $\downarrow$ CSTC (limbic) $\downarrow$ FPN $\uparrow$
(Göttlich et al. 2015)	17 (5 M, 12F; 32.6 ± 11.6y)	19 (4 M, 15F; 30.4 ± 9.6y)	Increased FC in the middle temporal gyrus and decreased FC in amygdala, hippocampus and ventral striatum	DMN $\uparrow$ CSTC (VMC) $\downarrow$
(Guo et al. 2022)	37 (22 M, 15F; 27.22 ± 8.6y)	37 (20 M, 17F; 24.16 ± 4.3y)	Decreased FC between cerebellum and FPN, limbic and SM networks in OCD	Cerebellum – FPN $\downarrow$ Cerebellum—CSTC (limbic) $\downarrow$ Cerebellum—CSTC (SM) $\downarrow$
(Gürsel et al. 2020)	49 (16 M, 33F; 34.42 ± 12.1y)	41 (19 M, 22F; 35.07 ± 10y)	Decreased FC between L and R-FPN and between the L-FPN and SN in OCD.	FPN $\downarrow$ FPN – SN $\downarrow$
(Han et al. 2023)	100 (53 M, 47F; 22.93 ± 9.3y)	106 (53 M, 53F; 23.09 ± 5.6y)	Decreased FC between DMN and CSTC (SM) in OCD.	DMN – CSTC (SM) $\downarrow$
(Harrison et al. 2009)	21 (10 M, 11F; 8.52 ± 5.9y)	21 (10 M, 11F; 26.2 ± 3.4y)	Significantly higher FC between the OFC and ventral caudate/NAc in OCD.	CSTC (VMC) $\uparrow$
(Harrison et al. 2013)	74 (42 M, 32F; 33.1 ± 8.3y)	74 (42 M, 32F; 32.7 ± 10.3y)	Increased FC between ventral caudate and OFC. Decreased FC between ventral caudate and BL insular cortex.	FPN – CSTC (VMC) $\uparrow$ FPN—SN $\downarrow$
(Haynes et al. 2018)	37 (16 M, 21F; 37.54 ± 9.9y)	37 (16 M, 21F; 34.03 ± 11.3y)	Decreased FC within the CSTC (limbic) in OCD.	CSTC (limbic) $\downarrow$
(Hong et al. 2018)	15 (7 M, 8F; 24.4 ± 5.4y)	15 (7 M, 8F; 22.5 ± 2.1y)	At baseline, increased FC between dACC-cingulate gyrus and decreased FC between dACC-superior frontal gyrus.	SN – DMN $\uparrow$ SN—FPN $\downarrow$
(Hou et al. 2013)	33 (18 M, 15F; 25.3 ± 9.6y)	33 (18 M, 15F; 25 ± 9.1y)	Significantly increased FC within the CSTC circuit (BL-OFC, ACC, caudate, putamen, thalamus, L-inferior frontal gyrus) and DMN (PCC). Increased FC in CSTC correlates with OCD severity.	CSTC (VMC) $\uparrow$ CSTC (SM) $\uparrow$ DMN $\uparrow$
(Hou et al. 2014)	39 (20 M, 19F; 26.6 ± 9.8y)	39 (20 M, 19F; 26 ± 6.3y)	Increased FC within caudate, OFC and middle temporal gyrus. Decreased FC within the VN and cerebellum in OCD compared to HC.	DMN $\uparrow$CSTC (DC) $\uparrow$ CSTC (VMC) $\uparrow$VN $\downarrow$ Cerebellum $\downarrow$
(Jang et al. 2010)	22 (16 M, 6F; 25.14 ± 6.96y)	22 (16 M, 6F; 24.36 ± 4.02y)	Decreased FC within the DMN regions in OCD compared to HC, indicating fronto-subcortical dysfunction.	DMN $\downarrow$
(Jia et al. 2020)	40 (27 M, 13F; 27.28 ± 8.2y)	38 (25 M, 13F; 27.18 ± 8.3y)	Decreased FC in OFC, thalamus, PCG, middle occipital gyrus in OCD.	CSTC (VMC) – CSTC (SM) $\downarrow$ CSTC (SM)—FPN $\downarrow$
(Jung et al. 2017)	61 (36 M, 25F; 25.64 ± 6.5y)	61 (41 M, 20F; 26.08 ± 7.2y)	Decreased FC between OFC and dorsomedial striatum (dorsal caudate). Increased FC between ventral striatum (NAc) and mPFC.	CSTC (VMC) – CSTC (DC) $\downarrow$ CSTC (VMC)—DMN $\uparrow$
(Kang et al. 2013)	18 (12 M, 6F; 24.9 ± 5.9y)	18 (12 M, 6F; 24.7 ± 2.7y)	Increased FC between caudate and middle cingulate cortex and PCG in OCD	CSTC (DC) – FPN $\uparrow$
(Kashyap et al. 2021)	20 (10 M, 10F; 28.8 ± 7y)	22 (10 M, 12F; 28.18 ± 6.7y)	Increased FC between cerebellar and VN nodes. Decreased FC between limbic CSTC and FPN.	Cerebellum – VN $\uparrow$ CSTC (limbic)—FPN $\downarrow$
(Kim et al. 2019)	102 (68 M, 34F; 25.3 ± 6.5y)	101 (62 M, 39F; 25.4 ± 6.9y)	Significantly larger FC within CSTC regions (temporal cortices, middle temporal gyrus, paracingulate gyrus, amygdala, hippocampus, putamen, thalamus)	CSTC (limbic) $\uparrow$ CSTC (SM) $\uparrow$
(Kinay et al. 2021)	15 (5 M, 10F; 15.27 ± 1.5y)	15 (5 M, 10F; 15.4 ± 1.4y)	Increased FC within the anterior DMN and decreased FC within the cerebellum and FPN in OCD.	DMN $\uparrow$ Cerebellum $\downarrow$ FPN $\downarrow$
(Koçak et al. 2012)	12 (6 M, 6F)	12 (6 M, 6F)	Higher connectivity within DMN regions (BL-IPL, L-vmPFC) in OCD.	DMN $\uparrow$
(Li et al. 2012)	20 (14 M, 6F; 28.2 ± 7y)	20 (14 M, 6F; 28.2 ± 7.3y)	Increased FC between R-anterior PFC and R-insula and middle cingulate cortex in OCD.	FPN $\uparrow$ FPN – SN $\uparrow$
(Li et al. 2018)	20 (13 M, 7F; 30.35 ± 7.5y)	20 (13 M, 7F; 30.55 ± 7.8y)	Increased FC between the L-DLPFC and R-OFC in OCD.	CSTC (DC) – CSTC (VMC) $\uparrow$
(Li et al. 2019)	45 (19 M, 26F; 28.2 ± 8.7y)	43 (20 M, 23F; 28.3 ± 8.3y)	Decreased FC between left thalamus-left orbital inferior frontal gyrus and R-thalamus-L inferior parietal gyrus in OCD.	CSTC (DC) – FPN $\downarrow$ CSTC (DC) $\downarrow$
(Li et al. 2020a)	88 (56 M, 32F; 29.16 ± 8.7y)	88 (56 M, 32F; 27.88 ± 10.6y)	Decreased FC between the R-DLPFC and R-OFC in OCD.	CSTC (DC) – CSTC (VMC) $\downarrow$ FPN—CSTC (VMC) $\downarrow$
(Li et al. 2020b)	42 (19 M, 23F; 27.21 ± 8.1y)	42 (19 M, 23F; 28.31 ± 8.4y)	Increased FC between L-ACC and R-middle temporal gyrus and between the middle temporal gyrus and cerebellum.	SN – DMN $\uparrow$ DMN—Cerebellum $\uparrow$
(Liu et al. 2021)	50 (26 M, 24F; 25.9 ± 3.6y)	50 (25 M, 25F; 23.7 ± 2y)	Decreased FC between cerebellum and FPN in OCD.	Cerebellum – FPN $\downarrow$
(Luo et al. 2021)	29 (19 M, 10F; 27.8 ± 9.4y)	40 (25 M, 15F; 27.9 ± 9.2y)	Increased FC within DMN and SN, with negative coupling between DMN and SN in OCD.	DMN $\uparrow$ SN $\uparrow$ DMN – SN $\downarrow$
(Lv et al. 2020)	40 (27 M, 13F; 27.28 ± 8.2y)	38 (25 M, 13F; 27.18 ± 8.3y)	Increased FC between cerebellum and mPFC, middle temporal gyrus in OCD.	Cerebellum – DMN $\uparrow$
(Lv et al. 2021)	40 (27 M, 13F; 27.28 ± 8.2y)	38 (25 M, 13F; 27.18 ± 8.3y)	Decreased FC in L-DLPFC, R-precuneus and L-PCG. Increased FC in L-thalamus and cerebellum.	CSTC (DC) – FPN $\downarrow$ CSTC (DC)—CSTC (SM) $\downarrow$ CSTC (SM)—Cerebellum $\uparrow$
(Lv et al. 2022)	40 (27 M, 13F; 27.28 ± 8.2y)	38 (25 M, 13F; 27.18 ± 8.3y)	Decreased FC within the PCG and increased FC between R-thalamus, caudate and L-inferior parietal lobule and cerebellum.	CSTC (SM) $\downarrow$ CSTC (DC) – DMN $\uparrow$
(Ma et al. 2022)	62 (31 M, 31F; 28.83 ± 7.4y)	60 (30 M, 30F; 30.95 ± 8.6y)	Decreased FC between the parahippocampal gyrus and PCG and superior temporal gyrus.	DMN $\downarrow$ DMN – CSTC (SM) $\downarrow$
(Meunier et al. 2012)	18 (11 M, 7F; 35.4 ± 9.8y)	18 (15 M, 3F; 32.7 ± 6.9y)	Decreased FC in R-OFC and between OFC and PCC in OCD	CSTC (VMC) $\downarrow$ CSTC (VMC) – DMN $\downarrow$
(Moody et al. 2017)	43 (22 M, 21F; 33 ± 10.7y)	24 (14 M, 10F; 31 ± 12y)	No difference in FC between OCD and HC at baseline.	-
(Moreira et al. 2017)	40 (13 M, 27F; 26.28 ± 6.6y)	40 (13 M, 27F; 26.45 ± 5.4y)	Decreased FC between OFC-ACC and lingual-PCG. Increased FC between thalamus-occipital lobe.	CSTC (VMC) – SN $\downarrow$ DMN—CSTC (SM) $\downarrow$ VN $\uparrow$
(Moreira et al. 2019)	40 (13 M, 27F; 26.52 ± 6.6y)	40 (13 M, 27F; 26.45 ± 5.4y)	Reduced FC within and between visual and SM networks and increased FC between DMN-Cerebellum in OCD.	VN $\downarrow$ CSTC (SM) $\downarrow$ CSTC (SM) – VN $\downarrow$ DMN—Cerebellum $\uparrow$
(Murayama et al. 2021)	47 (18 M, 29F; 33.3 ± 11.9y)	62 (22 M, 40F; 32.61 ± 11y)	Significantly higher FC between the cerebellum (R-lobule VI) and L-precuneus	Cerebellum – DMN $\uparrow$
(Nakamae et al. 2014)	20 (6 M, 14F; 35.3 ± 9.4y)	20 (9 M, 11F; 32.9 ± 6.9y)	Increased FC between the OFC and ventral striatum (NAc) in OCD	CSTC (VMC) $\uparrow$
(Naze et al. 2023)	52 (29 M, 23F; 30.2 ± 7.9y)	45 (27 M, 18F; 32.5 ± 8.7y)	Higher FC between the OFC and NAc, but lower FC between dorsal putamen and lateral-PFC	CSTC (VMC) $\uparrow$ CSTC (SM) – CSTC (DC) $\downarrow$
(Park et al. 2020)	23 (19 M, 4F; 27.74 ± 5.4y)	23 (19 M, 4F; 22.57 ± 3.5y)	Increased FC between the putamen and several cortical regions (PCG, angular gyrus) in OCD.	CSTC (SM) $\uparrow$ CSTC (SM) – DMN $\uparrow$
(Park et al. 2022)	107 (72 M, 35F; 25.2 ± 2.1y)	110 (69 M, 41F; 25 ± 4.9y)	Decreased FC within the CSTC (VMC) and increased FC within the DMN.	CSTC (VMC) $\downarrow$ DMN $\uparrow$
(Peng et al. 2014b)	15 (10 M, 5F; 26.7 ± 4.8y)	28 (21 M, 7F; 27.5 ± 8.4y)	Significantly reduced FC within the DMN (PCC) and in OCD and increased FC with AI, R-inferior frontal lobe.	DMN $\downarrow$ DMN – SN $\uparrow$
(Peng et al. 2014a)	30 (21 M, 9F; 28 ± 6.8y)	30 (22 M, 8F; 27.3 ± 8.2y)	Decreased FC within the DMN and increased FC between CSTC-SM and FPN.	DMN $\downarrow$ CSTC (SM) – FPN$\uparrow$
(Peng et al. 2022)	62 (45 M, 17F; 26.8 ± 8.3y)	73 (51 M, 22F; 27.2 ± 9.4y)	Significantly higher FC within the caudate (CSTC-DC) in OCD.	CSTC (DC) $\uparrow$
(Picó-Pérez et al. 2019)	73 (43 M, 30F; 37.74 ± 10.19y)	42 (22 M, 20F; 39.43 ± 9.79y)	Reduced connectivity between R-amygdala and R-post central gyrus in OCD, significantly correlated to OCD severity.	CSTC (SM) – CSTC (limbic) $\downarrow$
(Ping et al. 2013)	20 (16 M, 4F; 27.1 ± 8y)	20 (16 M, 4F; 27.6 ± 8.2y)	Increased FC between OFC and ventral ACC in OCD.	CSTC (VMC) – SN $\uparrow$
(Posner et al. 2014)	23 (11 M, 12F; 30.9 ± 8.8y)	20 (11 M, 9F; 32.6 ± 10y)	Reduced FC within the limbic CSTC loop in unmedicated OCD compared to HC, positively correlated to OCD severity.	CSTC (limbic) $\downarrow$
(Posner et al. 2017)	30 (16 M, 14F; 29.1 ± 7.9y)	32 (16 M, 16F; 27.9 ± 8y)	Significantly increased FC between vmPFC and AI in OCD.	DMN – SN $\uparrow$
(Pujol et al. 2019)	160 (86 M, 74F; 35.41 ± 9.7y)	121 (66 M, 55F; 34.6 ± 10.2y)	Decreased FC between BL-SM cortex, BL-visual cortex, L-AI and BL-OFC in OCD.	CSTC (SM) – VN$\downarrow$ CSTC (SM)—SN$\downarrow$ CSTC (SM)—CSTC (VMC) $\downarrow$
(Raposo-Lima et al. 2022)	75 (32 M, 43F; 26 ± 11y)	71 (24 M, 47F; 25 ± 6y)	Increased FC within the FPN and decreased FC within the visual network in OCD.	FPN $\uparrow$ VN $\downarrow$
(Reess et al. 2016)	41 (14 M, 27F; 32.5 ± 10y)	42 (18 M, 24F; 31.8 ± 8.3y)	Decreased FC between OFC-putamen, amygdala-AI in OCD.	CSTC (VMC) – CSTC (SM) $\downarrow$ CSTC (limbic)—SN $\downarrow$
(Sakai et al. 2011)	20 (8 M, 12F; 30.9 ± 9.3y)	23 (10 M, 13F; 30.8 ± 7.7y)	Increased FC between ventral striatum and OFC, DLPFC and vmPFC in OCD.	CSTC (VMC) $\uparrow$ CSTC (VMC) – CSTC (DC) $\uparrow$ CSTC (VMC)—CSTC (limbic) $\uparrow$
(Sha et al. 2020b)	44 (15 M, 29F; 23.61 ± 4.8y)	43 (18 M, 25F; 23.51 ± 4.1y)	Significantly higher FC between L-caudate and BL-DLPFC in OCD.	CSTC (DC) $\uparrow$ FPN $\uparrow$
(Sha et al. 2020a)	44 (15 M, 29F; 23.61 ± 4.8y)	43 (18 M, 25F; 23.51 ± 4.1y)	Decreased FC within the CSTC-SM and increased FC between CSTC-SM and cerebellum in OCD.	CSTC (SM) $\downarrow$ CSTC (SM) – Cerebellum $\uparrow$
(Shan et al. 2019)	20 (7 M, 13F; 33.4 ± 5.8y)	20 (9 M, 11F; 35.2 ± 5.3y)	Decreased FC in the L-middle temporal gyrus in OCD	DMN $\downarrow$
(Shi et al. 2021)	41 (21 M, 20F; 29.1 ± 7.3y)	36 (18 M, 18F; 29.7 ± 7.5y)	Decreased FC between nodes of the SN and DMN with increased FC within nodes of the DMN	SN – DMN $\downarrow$ DMN $\uparrow$
(Shin et al. 2014)	25 (17 M, 8F; 26.3 ± 6.2y)	23 (13 M, 10F; 26.9 ± 5.5y)	Decreased FC within the FPN and between FPN and VN at baseline.	FPN $\downarrow$ FPN – VN $\downarrow$
(Stern et al. 2012)	30 (15 M, 15F; 25.8 ± 6.7y)	32 (15 M, 17F; 28.35 ± 8.5y)	Greater FC within the FPN and between FPN and DMN nodes (PCC, inferior parietal lobe, dmPFC) in OCD	FPN $\uparrow$ FPN – DMN $\uparrow$
(Takagi et al. 2017)	56 (23 M, 33F; 32.64 ± 9.6y)	52 (26 M, 26F; 29.4 ± 7.5y)	Increased FC in OCD within FPN and DMN in OCD compared to HC.	FPN $\uparrow$ DMN $\uparrow$
(Tang et al. 2023)	40 (25 M, 15F; 29.7 ± 9.4y)	57 (32 M, 25F; 28.7 ± 8.9y)	Increased FC between PCC-precuneus and decreased FC between frontal-middle cingulate gyrus.	DMN $\uparrow$ FPN $\downarrow$
(Tian et al. 2016)	29 (21 M, 8F; 26.6 ± 8.1y)	29 (21 M, 8F; 26.1 ± 7.9y)	Increased FC in OCD than HC, distributed within the CSTC circuits (Brain hubs found at OFC, mPFC, DLPFC, ACC, PCC, insula). Significantly correlated with OCD symptom severity.	CSTC (VMC) – CSTC (DC) $\uparrow$ CSTC (DC)—CSTC (limbic) $\uparrow$ Cerebellum $\uparrow$
(Tikoo et al. 2020)	10 (7 M, 3F; 10.9 ± 2.5y)	11 (2 M, 9F; 9.9 ± 1.3y)	Increased FC in within nodes of CSTC (SM), DMN, FPN, SN in OCD.	CSTC (SM) $\uparrow$DMN $\uparrow$ FPN $\uparrow$SN $\uparrow$
(Tikoo et al. 2021)	11 (7 M, 4F; 10.7 ± 2.5y)	12 (3 M, 9F; 10 ± 1.2y)	Decreased FC between cerebellum (dentate nucleus) and L-PCG, L-inferior temporal gyrus and L-crus II in OCD.	Cerebellum $\downarrow$ Cerebellum – CSTC (SM) $\downarrow$ Cerebellum—FPN $\downarrow$
(Tomiyama et al. 2019)	37 (16 M, 21F; 33.49 ± 11.4y)	40 (17 M, 23F; 35.48 ± 11.1y)	Increased FC between dorsal caudate to dorsal-ACC and AI in OCD	CSTC (DC) – SN $\uparrow$
(Tomiyama et al. 2022b)	41 (16 M, 25F; 33.34 ± 11.7y)	49 (19 M, 31F; 33.33 ± 10.4y)	Increased FC between pre-SMA and IFG, BL-inferior parietal lobule, dACC and AI in OCD.	CSTC (SM) – FPN $\uparrow$ CSTC (SM)—DMN $\uparrow$ CSTC (SM)—SN $\uparrow$
(Tomiyama et al. 2022a)	47 (18 M, 29F; 33.3 ± 11.9y)	62 (22 M, 40F; 32.61 ± 11y)	Increased FC between AI and PCC and within the CSTC (DC).	SN – DMN $\uparrow$ CSTC (DC) $\uparrow$
(Vaghi et al. 2017)	44 (21 M, 23F; 36.14 ± 10.7y)	43 (22 M, 21F; 37.51 ± 12.1y)	Reduced FC between the caudate and vlPFC, and the putamen and DLPFC in OCD. Hyperconnectivity between basal ganglia and cerebellum	CSTC (SM) – CSTC (DC) $\downarrow$ CSTC (DC)—CSTC (limbic) $\downarrow$ CSTC (DC)—cerebellum $\uparrow$
(Versace et al. 2019)	48 (18 M, 30F; 23.3 ± 4.5y)	45 (17 M, 28F; 23.2 ± 3.8y)	Decreased FC between ACC and mPFC in OCD.	SN – DMN $\downarrow$
(Wang et al. 2019)	22 (11 M, 11F; 22.41 ± 6.2y)	22 (11 M, 11F; 22.68 ± 2.3y)	Increased FC between subregions of DMN and FPN. Decreased FC within DMN.	DMN – FPN $\uparrow$ DMN $\downarrow$
(Weber et al. 2014)	11 (6 M, 6F; 13 ± 2.9y)	9 (5 M, 4F; 12.7 ± 3.2y)	Decreased FC between ACC, BL-DLPFC and increased FC within auditory network in OCD.	SN – CSTC (DC) $\downarrow$ DMN $\uparrow$
(Xia et al. 2020)	40 (22 M, 18F; 22.48 ± 6.1y)	42 (21 M, 21F; 22.76 ± 6.1y)	Increased FC within the SN (BL-AI and ACC) and BL-AI with the DMN	SN $\uparrow$ SN – DMN $\uparrow$
(Xie et al. 2017)	68 (37 M, 31F; 30.24 ± 7.8y)	33 (17 M, 16F; 25.61 ± 7.4y)	Increased FC between dACC, caudate and AI. Decreased FC within FPN.	SN $\uparrow$FPN $\downarrow$ SN – CSTC (DC) $\uparrow$
(Xing et al. 2020)	61 (16 M, 45F; 26.1 ± 8.1y)	67 (23 M, 44F; 21.3 ± 5y)	Significantly lower FC within the cerebellum and between the cerebellum and inferior occipital cortex and thalamus in OCD.	Cerebellum $\downarrow$ Cerebellum – VN $\downarrow$
(Xu et al. 2019)	27 (16 M, 11F; 29.22 ± 8.1y)	21 (12 M, 9F; 33.57 ± 7.2y)	Decreased FC between cerebellum and several networks including DMN, affective-limbic and sensorimotor networks in OCD.	DMN – cerebellum $\downarrow$ CSTC (limbic)—cerebellum $\downarrow$ CSTC (SM)—cerebellum $\downarrow$
(Xu et al. 2021)	36 (22 M, 14F; 29.14 ± 7.7y)	50 (27 M, 23F; 30.2 ± 7.2y)	Increased FC of caudate-OFC, ventral striatum (VS)-OFC, VS-mPFC, and putamen-SMA, and decreased FC of caudate-ACC, putamen-ACC, and putamen- DLPFC.	CSTC (VMC) $\uparrow$, CSTC (SM) $\uparrow$ CSTC (VMC)—CSTC (DC) $\uparrow$ CSTC (VMC)—CSTC (limbic) $\uparrow$ CSTC (DC)—SN $\downarrow$ CSTC (SM)—SN $\downarrow$ CSTC (SM)—FPN $\downarrow$
(Xu et al. 2022)	40 (30 M, 10F; 24.63 ± 7.8y)	43 (18 M, 25F; 24.16 ± 4.3y)	Decreased FC between dmPFC-SMA and IFG-OFC in OCD.	CSTC (DC) – CSTC (SM) $\downarrow$ FPN—CSTC (VMC) $\downarrow$
(Xu et al. 2023a)	100 (52 M, 48F; 23.15 ± 9.3y)	120 (57 M, 63F; 22.17 ± 5.9y)	Increased FC between R-AI to L-DLPFC, R-DLPFC to cerebellum, cerebellum to PCC and ACC. Decreased FC between L-AI to L-DLPFC, R-AI to ACC and within R-DLPFC.	SN – FPN $\uparrow$ FPN—Cerebellum $\uparrow$ Cerebellum—DMN $\uparrow$ SN $\downarrow$FPN $\downarrow$
(Xu and Zhang 2023)	103 (54 M, 49F; 19 ± 13y)	118 (56 M, 62F; 22 ± 8y)	Decreased FC within the cerebellum, CSTC-SM, DMN in OCD compared to HC.	Cerebellum$\downarrow$ CSTC (SM) $\downarrow$ DMN$\downarrow$
(Xu et al. 2023b)	73 (39 M, 34F; 22.7 ± 8.6y)	54 (30 M, 24F; 22.28 ± 6.8y)	Increased FC between angular gyrus and inferior parietal lobule (DMN), and between L-middle occipital gyrus (FPN) and cerebellum.	DMN $\uparrow$ FPN – Cerebellum $\uparrow$
(Yang et al. 2019)	68 (45 M, 23F; 27.99 ± 8.2y)	68 (45 M, 23F; 27.57 ± 8.6y)	Significantly higher FC within the limbic CSTC and lower FC between putamen and SMA in OCD	CSTC (limbic) $\uparrow$ CSTC (SM) $\downarrow$
(Ye et al. 2020)	73 (38 M, 35F; 28.93 ± 5.3y)	79 (42 M, 37F; 27.73 ± 5.7y)	Increased FC within the CSTC (SM) in PCG and decreased FC within SN (L-AI) in OCD.	CSTC (SM) $\uparrow$ SN $\downarrow$
(Ye et al. 2021)	73 (38 M, 35F; 28.93 ± 5.3y)	79 (42 M, 37F; 27.73 ± 5.7y)	Decreased FC between the precuneus and vermis of the cerebellum in OCD	FPN – Cerebellum $\downarrow$
(Yu et al. 2022)	45 (26 M, 19F; 28.19 ± 7.9y)	45 (22 M, 23F; 25.91 ± 3.9y)	Increased FC between L-medial superior frontal gyrus and R-caudate in OCD.	FPN – CSTC (DC) $\uparrow$
(Yun et al. 2017)	24 (17 M, 7F; 24.9 ± 6.7y)	34 (24 M, 10F; 24 ± 4.1y)	Significantly higher FC within R-ACC and L-DLPFC in OCD compared to HC	SN – FPN $\uparrow$
(Zhang et al. 2011)	18 (14 M, 4F; 23.3 ± 5y)	16 (12 M, 4F; 24.1 ± 5.4y)	Increased FC between Mid-cingulate and PCC and within cerebellum. Decreased FC between AI and Post temporal cortex.	Cerebellum $\uparrow$ FPN – DMN $\uparrow$ SN—DMN $\downarrow$
(Zhang et al. 2017)	23 (15 M, 8F; 32.09 ± 10.6y)	23 (15 M, 8F; 31.39 ± 10y)	Decreased FC of ACC-DLPFC and increased FC between dACC and caudate in OCD.	SN – FPN $\downarrow$ SN—CSTC (DC) $\uparrow$
(Zhang et al. 2019)	30 (14 M, 16F; 27.4 ± 8.9y)	26 (10 M, 16F; 27.8 ± 10.2y)	Weakened FC between regions of CSTC and cerebellum (L-crus II, lobule VIII, R-striatum, cingulate) in OCD.	CSTC (SM) – Cerebellum $\downarrow$
(Zhang et al. 2021)	58 (37 M, 21F; 27.2 ± 6.6y)	72 (34 M, 38F; 24.4 ± 3.4y)	Increased FC between L-caudate and BL-SMA in OCD.	CSTC (DC) – CSTC (SM) $\uparrow$
(Zhao et al. 2021)	51 (32 M, 19F; 27.25 ± 7.5y)	25 (17 M, 8F; 25.92 ± 5.1y)	Decreased FC within the limbic CSTC loop and increased FC between SMA-putamen.	CSTC (limbic)$\downarrow$ CSTC (SM) $\uparrow$
(Zhou et al. 2022)	85 (52 M, 33F; 29.18 ± 8.7y)	85 (51 M, 34F; 28.16 ± 10.9y)	Increased FC between BL-AI and BL-precuneus extending to SMA. Decreased FC between R-AI and lingual gyrus.	SN – FPN$\uparrow$ SN—CSTC (SM) $\uparrow$ SN—DMN $\downarrow$

*Note.* OCD—obsessive-compulsive disorder, fMRI—functional magnetic resonance imaging, M—male, F—female, y—years old, SD—standard deviation, NAc—nucleus accumbens, mPFC—medial prefrontal cortex, lPFC—lateral prefrontal cortex, CSTC—cortico-striato-thalamo-cortical, VMC—ventral motivational circuit, dACC—dorsal anterior cingulate cortex, l—left, r—right, BL—bilateral, DLPFC—dorsolateral prefrontal cortex, FPN—frontoparietal network, vmPFC—ventromedial prefrontal cortex, OFC—orbitofrontal cortex, SM—sensorimotor, DC—dorsal cognitive, OCPD—obsessive-compulsive personality disorder, PCC—posterior cingulate cortex, VN—visual network, SMA—supplementary motor area, AI—anterior insula.

Within EEG studies reviewed, only two reported FC findings related to FC within large-scale brain networks (Choi et al. 2021; Olbrich et al. 2013). Choi et al. (2021) reported significantly lower FC within the DMN, while Olbrich et al. (2013) reported no difference in FC within the DMN in OCD when compared to HC. This finding is consistent with the results from fMRI studies discussed later, where decreased FC within the DMN was found to be a major finding in resting-state fMRI studies of OCD (Beucke et al. 2014; Fan et al. 2023; Wang et al. 2019).

### Resting-state fMRI studies

**Fig. 2:**
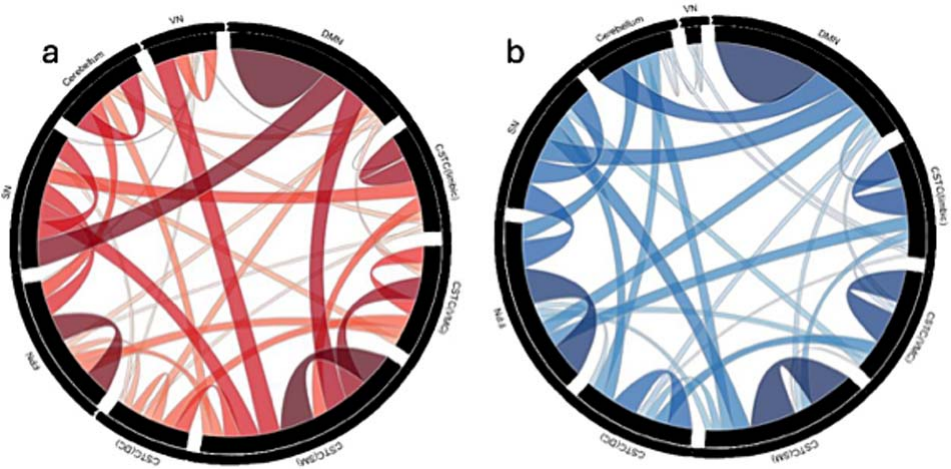
a) Chord diagram illustrating decreased functional connectivity within and between large-scale brain networks. b) Chord diagram illustrating increased functional connectivity within and between large-scale brain networks. In both figures, the connected network is indicated by the two edges of each line. The thickness of the lines relates to the cumulative number of OCD participants providing support for each finding. (DMN—Default mode network, CSTC—Cortico-striato-thalamo-cortical circuit, VMC—Ventral motivational circuit, SM—Sensorimotor circuit, DC—Dorsal cognitive circuit, FPN—Fronto-parietal network, SN—Salience network, VN—Visual network).

Table 2 summarizes key findings of resting-state fMRI studies. Figure 2 presents the chord diagrams depicting findings of the quantitative analysis described in Section 2.4. The values used to create the diagrams are presented in Supplementary Table S2. In the decreased FC section (Fig. 2a), the most consistently reported finding across studies was within the DMN, with a total of 525 OCD participants across 10 studies supporting this conclusion. This was followed by decreased FC within the sensorimotor CSTC circuit, a finding supported by a total of 381 OCD participants across 7 studies. Within the analyses of inter-network FC, the most consistent finding was decreased inter-network FC between the DMN and SN with a total of 285 OCD participants across 7 studies providing evidence for this result. As presented in Fig. 2b, the most consistent findings among studies that reported increased intra-network FC was within the DMN with a total of 528 participants across 15 studies, followed by increased FC within the sensorimotor CSTC with a total of 458 participants across 10 studies. The most consistent finding of increased inter-network FC was found between the DMN-cerebellum with a total of 269 OCD participants across 5 studies.

### Task-related fMRI studies

Table 3 summarizes participant data, task paradigms and key findings of task-related fMRI studies in OCD. The most consistently reported finding among task-related studies was an increase in FC within the FPN, observed in a total of 177 OCD patients across three studies that used set shifting or task switching paradigms. The most consistent finding among studies reporting decreased FC was observed between the limbic CSTC circuit and the DMN, involving a total of 59 OCD participants across two studies.

**Table 3:** Task-related fMRI connectivity studies in OCD.

**Author (year)**	**OCD sample (Male, Female, Mean age, SD)**	**Comparison sample (Male, Female, Mean age, SD)**	**fMRI methodology**	**Key findings**	**Involved neurocircuitry and direction of connectivity findings**
(Admon et al. 2012)	13 (10 M, 3F; 25.5 ± 1y)	13 (10 M, 3F; 27 ± 0.5y)	fMRI collected during an interactive risky choice task	Reduced FC between amygdala-NAc and OFC-dACC in OCD.	CSTC (limbic) – CSTC (VMC) $\downarrow$ CSTC (VMC)—SN $\downarrow$
(Agam et al. 2014)	21 (8 M, 13F; 33 ± 11y)	20 (11 M, 9F; 33 ± 11y)	fMRI collected during an anti-saccade task	Increased FC between the PCC and ACC in OCD.	DMN – SN $\uparrow$
(Alves-Pinto et al. 2019)	39 (14 M, 23F; 33.7 ± 10.1y)	37 (16 M, 23F; 32.2 ± 8.3y)	fMRI collected during a reward learning task	Stronger effective and reward-related connectivity between vmPFC, L- and R-OFC, suggestive of dysfunction in parts of both DMN and CSTC networks.	DMN – CSTC (VMC) $\uparrow$
(Cardoner et al. 2011)	21 (10 M, 11F; 28.52 ± 5.9y)	21 (10 M, 11F; 26.2 ± 3.4y)	fMRI collected during an emotional face-processing task	Increased FC between amygdala and DLPFC during task.	FPN – CSTC (limbic) $\uparrow$
(Cocchi et al. 2012)	17 (8 M, 9F; 32.8 ± 10.8y)	19 (10 M, 9F; 30.6 ± 7.2y)	fMRI collected during a multisource inference task	Significantly higher FC within the SN (dACC-AI) in OCD.	SN $\uparrow$
(de Vries et al. 2014)	43 (22 M, 21F; 38.1 ± 9.7y)	37 (17 M, 20F; 39.2 ± 11.5y)	fMRI collected during a visuo-spatial n-back task	Increased FC between L-SMA and BL-amygdala, L-DLPFC and R-amygdala in OCD.	CSTC (SM) – CSTC (limbic) $\uparrow$ FPN—CSTC (limbic) $\uparrow$
(Figee et al. 2013)	16 (9 M, 7F; 45 ± 9.7y)	13 (7 M, 7F; 45 ± 9.2y)	fMRI collected during a reward anticipation task	Hyperconnectivity in the frontostriatal networks in OCD is normalized with DBS to the NAc (particularly between NAc and mPFC and lPFC)	CSTC (VMC) – CSTC (limbic) $\uparrow$
(Fitzgerald et al. 2010)	18 (6 M, 12F; 13.9 ± 2.6y)	18 (6 M, 12F; 14.1 ± 2.6y)	fMRI collected during a performance monitoring task and at rest.	Increased task-related dACC -vmPFC FC and decreased resting dACC-R operculum and vmPFC-PCC FC in OCD.	SN $\downarrow$ SN – CSTC (limbic) $\uparrow$ CSTC (limbic)—DMN $\downarrow$
(Fontenelle et al. 2012)	11 (7 M, 4F; 36.3 ± 7.9y)	10 (6 M, 4F; 34.8 ± 7y)	fMRI collected during a sad mood induction task	Increased FC between ACC and ventral caudate and NAc in OCD	SN – FPN $\uparrow$ SN—CSTC (VMC) $\uparrow$
(Hampshire et al. 2020)	20 (17 M, 3F; 37.6 ± 14.6y)	20 (15 M, 5F; 36.3 ± 8.3y)	fMRI collected during a stop signal task	Reduced FC between cerebellum-FPN and cerebellum-DMN in OCD	Cerebellum – FPN $\downarrow$ Cerebellum—DMN $\downarrow$
(Han et al. 2016)	20 (12 M, 8F; 25.5 ± 5.4y)	21 (14 M, 7F; 22.57 ± 4.5y)	fMRI collected during a delayed-response working memory task	Emotional distraction significantly reduces FC between DLPFC-OFC in OCD compared to HC.	FPN – CSTC (VMC) $\downarrow$
(Jaspers-Fayer et al. 2022)	23 (9 M, 14F; 15.1 ± 2.6y)	23 (7 M, 16F; 14.2 ± 3.1y)	fMRI collected during a Tower of London task	Increased FC between superior/middle frontal gyrus and precuneus/inferior parietal lobule.	FPN – DMN $\uparrow$
(Jhung et al. 2014)	26 (20 M, 6F; 27.25 ± 6.1y)	18 (15 M, 3F; 28.2 ± 6.6y)	fMRI collected during a contamination provocation task	Contamination group showed significantly higher FC between ventral striatum (NAc) and R-insula.	CSTC (VMC) – SN $\uparrow$
(Jung et al. 2013)	19 (12 M, 7F; 25.84 ± 7.15y)	18 (11 M, 7F; 24.83 ± 3.88y)	fMRI collected at rest and during a monetary incentive delay task	Resting state – increased FC between NAc and lateral OFC Incentive processing—decreased FC between NAc and areas of the limbic CSTC (amygdala)	Rest: CSTC (VMC) $\uparrow$ Task: CSTC (VMC)—CSTC (limbic) $\downarrow$
(Kim et al. 2020)	17 (12 M, 5F; 26.4 ± 6y)	21 (11 M, 10F; 26 ± 5.3y)	fMRI collected during a Tower of London task	At baseline, OCD group showed decreased FC between FPN and DMN.	FPN – DMN $\downarrow$
(Kim et al. 2022)	105 (70 M, 35F; 25.05 ± 6.6y)	99 (64 M, 35F; 23.93 ± 5.8y)	fMRI collected during a set shifting task.	Increased FC within the bilateral inferior-middle frontal gryus and between the anterior caudate-thalamus.	FPN $\uparrow$ CSTC (DC) $\uparrow$
(Koch et al. 2018)	44 (17 M, 27F; 32.7 ± 9.3y)	37 (15 M, 22F; 32 ± 8y)	fMRI collected during a monetary reward task	Increased connectivity between PCC and vmPFC (areas of the DMN) in OCD compared to HC.	DMN $\uparrow$
(Lee et al. 2022)	41 (36 M, 5F; 25.27 ± 6.5y)	47 (46 M, 1F; 22.59 ± 1.9y)	fMRI collected during a thought-action fusion task	Decreased FC between Midcingulate cortex-AI, Middle temporal gyrus-amygdala, AI-precuneus in OCD	FPN – SN $\downarrow$ DMN—CSTC (limbic) $\downarrow$
(Liu et al. 2023)	42 (25 M, 17F; 21.86 ± 4.9y)	48 (21 M, 27F; 20.65 ± 2.1y)	fMRI collected during a cued task switching paradigm	Increased FC within the FPN and between FPN and DMN in OCD.	FPN $\uparrow$ FPN – DMN $\uparrow$
(Marsh et al. 2014)	22 (11 M, 11F; 30 ± 9.1y)	22 (11 M, 11F; 30.14 ± 9.4y)	fMRI collected during a Simon Spatial Incompatibility task	Increased FC between putamen and SFG, inferior parietal lobule and caudate in OCD.	CSTC (SM) – FPN $\uparrow$ CSTC (SM)—DMN $\uparrow$ CSTC (SM)—CSTC (DC) $\uparrow$
(Paul et al. 2019)	21 (8 M, 13F; 33.1 ± 10.8y)	21 (8 M, 13F; 33.1 ± 10.1y)	fMRI collected during a symptom provocation task	Reduced FC between L-OFC and amygdala in OCD during symptom provocation.	CSTC (VMC) – CSTC (limbic) $\downarrow$
(Picó-Pérez et al. 2022)	30 (13 M, 17F; 28.97 ± 11.14y)	29 (14 M, 15F; 29.35 ± 12.14y)	fMRI collected during a cognitive reappraisal task	Increased FPN connectivity (between left angular gyrus and left vlPFC) in OCD compared to HC during an emotion regulation task, and decreased FC in OCD when experiencing negative emotions.	FPN $\uparrow$
(Ravindran et al. 2020)	31 (13 M, 18F; 34 ± 8.5y)	17 (9 M, 8F; 32.6 ± 9.2y)	fMRI collected during an emotion provocation task	Increased FC between PCC and visual cortices and CSTC regions. Checking subtypes—motor cortices, washing subtypes—anterior insula and OFC.	DMN—VN $\uparrow$ DMN—CSTC (limbic) $\uparrow$
(Rus et al. 2017)	42 (15 M, 27F; 32.5 ± 10y)	37 (15 M, 22F; 30.99 ± 7.6y)	fMRI collected during a contamination provocation task	Increased FC between L-amygdala and parietal cortex in OCD.	CSTC (limbic) – DMN $\uparrow$
(Schlösser et al. 2010)	21 (5 M, 16F; 31.3 ± 10.2y)	21 (5 M, 16F; 28.8 ± 8.3y)	fMRI collected during a modified Stroop task	Significantly higher FC between dACC and l-DLPFC in OCD.	DMN – FPN $\uparrow$
(Stern et al. 2011)	39 (17 M, 22F; 27.8 ± 8.7y)	38 (18 M, 20F; 28.9 ± 9.1y)	fMRI collected during an incentive flanker task	Increased FC between vmPFC and AI, R-thalamus in OCD	CSTC (limbic) – SN $\uparrow$
(Stern et al. 2017)	18 (7 M, 11F; 28.2 ± 7.1y)	18 (8 M, 10F; 27.2 ± 6.5y)	fMRI collected during internal/external attentional tasks.	Stronger FC between dmPFC and occipital regions in OCD	DMN – VN$\uparrow$
(Thorsen et al. 2020)	31 (12 M, 19F; 30.19 ± 9.2y)	26 (8 M, 18F; 31 ± 10.7y)	fMRI collected during a stop signal task	Increased FC between R-amygdala and R-inferior frontal gyrus and pre-SMA.	CSTC (limbic) – FPN $\uparrow$ CSTC (limbic)—CSTC (SM) $\uparrow$
(Vaghi et al. 2017)	21 (3 M, 18F; 37.9 ± 14.3y)	20 (5 M, 15F; 36.45 ± 8.5y)	fMRI collected during a Tower of London task	OCD group showed reduced FC between R-DLPFC and putamen.	FPN – CSTC (SM) $\downarrow$
(van der Straten et al. 2020)	23 (10 M, 13F; 33.48 ± 2y)	23 (12 M, 11F; 33.52 ± 3.1y)	fMRI collected during a stress induction task	Stress induction caused significant reduction in FC between the caudate and precuneus in OCD.	CSTC (DC) – DMN $\downarrow$
(van Velzen et al. 2015)	41 (21 M, 20F; 38.6 ± 9.8y)	37 (18 M, 19F; 39.7 ± 11.6y)	fMRI collected during a stop signal task	Decreased FC between inferior frontal gyrus and amygdala in OCD.	FPN – CSTC (limbic) $\downarrow$

*Note*. OCD—obsessive-compulsive disorder, fMRI—functional magnetic resonance imaging, M—male, F—female, y—years old, SD—standard deviation, NAc—nucleus accumbens, mPFC—medial prefrontal cortex, lPFC—lateral prefrontal cortex, CSTC—cortico-striato-thalamo-cortical, VMC—ventral motivational circuit, dACC—dorsal anterior cingulate cortex, l—left, r—right, DLPFC—dorsolateral prefrontal cortex, FPN—frontoparietal network, vmPFC—ventromedial prefrontal cortex, OFC—orbitofrontal cortex, SM—sensorimotor, DC—dorsal cognitive, OCPD—obsessive-compulsive personality disorder, PCC—posterior cingulate cortex, VN—visual network.

## Discussion

In this review, findings from both EEG and fMRI FC studies in OCD were meticulously examined and synthesized. Within EEG studies, significantly lower FC was consistently observed within the delta and alpha frequency bands in OCD groups compared to HC, while results in the theta and beta bands were less conclusive. Contradictory reports emerged from resting-state fMRI studies, revealing both higher and lower FC within the DMN and the sensorimotor CSTC circuit. Notably, many studies reported lower connectivity between the DMN and SN at rest, suggesting potential impairments of the TNM underlying OCD symptoms. Task-related hyperconnectivity between the DMN-SN and hypoconnectivity between the SN-FPN further suggest cognitive inflexibility in OCD, possibly stemming from dysfunction within the TNM.

### Key findings from EEG studies

Our review identified 10 EEG studies which measured FC at-rest in OCD. The majority of these studies (9 out of 10) reported significantly reduced connectivity in OCD groups compared to HC (Choi et al. 2021; Olbrich et al. 2013; Perera et al. 2023b; Saifutdinova et al. 2016; Tan et al. 2019; Tan et al. 2022; Velikova et al. 2010; Yazdi-Ravandi et al. 2018; Özçoban et al. 2018). Interpreting the results was challenging due to the limited number of studies. Slow delta band oscillations provide important information on both motivational and cognitive processes such as memory, attention, decision-making and planning (Sauseng and Klimesch 2008). Impaired FC within the delta band (Perera et al. 2023b; Özçoban et al. 2018) is related to the subcortical centres generating delta band activity such as the medial prefrontal cortex (mPFC) and orbitofrontal cortex (Murphy et al. 2009b), both of which are vital areas of the CSTC circuits that are implicated in the pathophysiology of OCD (Milad and Rauch 2012). Therefore, lower delta FC within CSTC circuitry may reflect a pathophysiological marker that potentially underpins the clinical symptoms of OCD.

Additionally, it is noteworthy that the only study focusing on theta band FC reported a significant increase in fronto-occipital FC (Desarkar et al. 2007). Research has found significant positive correlation between increased EEG coherence and increased subcortical metabolic activity (Newton et al. 1993). Furthermore, there is strong neuroimaging evidence of heightened metabolic activity in subcortical structures such as the basal ganglia and thalamus in OCD groups (Saxena and Rauch 2022). Therefore, the higher theta coherence may reflect overactivity within subcortical circuitry in OCD. However, this study used coherence as the sole connectivity measure, and did not follow optimal EEG connectivity analysis methods highlighted in a recent guideline (Miljevic et al. 2021). Therefore, further research is required to arrive at reliable conclusions regarding theta band connectivity in OCD.

Lower FC within the alpha band compared to HC was reported in 4 out of 10 studies (Choi et al. 2021; Tan et al. 2019; Tan et al. 2022; Velikova et al. 2010). One study that utilized source localisation found decreased alpha connectivity localized mainly within the DMN, predominantly connections involving the posterior cingulate cortex (PCC) (Choi et al. 2021). Research suggests that alterations in PCC activity may contribute to perseveration of obsessive thoughts and rumination commonly observed in OCD patients (Makovac et al. 2020). Furthermore, previous reports indicate that gray matter volume and resting-state metabolism within the PCC are higher in OCD groups compared to HC (Brennan et al. 2016; Hou et al. 2013). Consequently, it might be hypothesized that impaired FC within the PCC could lead to excessive obsessive rumination, while the observed increases in gray matter volume and metabolism might serve as compensatory mechanisms to mitigate this effect. However, in contrast to our findings of lower alpha band FC in OCD, previous research on major depressive disorder has reported significantly increased alpha band FC. (Miljevic et al. 2023). This suggests that the decreased alpha FC shown in OCD is unlikely to be attributed to comorbid depression but may instead represent a finding specifically associated with OCD.

Beta FC was also found to be significantly lower in OCD compared to HC (Olbrich et al. 2013; Yazdi-Ravandi et al. 2018). Beta band connectivity is associated with memory encoding, retrieval and maintenance of information (Palva et al. 2010). In this context, decreased connectivity within the beta band in the frontal brain regions during high vigilance states in OCD (Olbrich et al. 2013) may be related to previously reported dysfunction within these cognitive domains in individuals with OCD (Deckersbach et al. 2000). In contrast, one study reported increased beta FC in the OCD group during the eyes-open state (Tan et al. 2019). The authors attributed this finding to impaired suppression of recurrent, unwanted thoughts leading to excessive stress and anxiety (Vul et al. 2009). Furthermore, their findings were thought to provide evidence towards the abnormal small-world architecture within OCD patients. Small-world architecture refers to the efficient organization of neural connections that enables both specialized processing within local brain regions and rapid communication between distant brain regions (Bassett and Bullmore 2006). Tan et al. (2019) found elevated short-range beta FC within occipital regions and reduced alpha FC in long-range connections, supporting this finding (Tan et al. 2019). However, it is known that beta band findings are highly prone to muscle artifacts (Muthukumaraswamy 2013). Therefore, if muscle artifacts were not sufficiently controlled for by the EEG pre-processing, this confound may have contributed to the contradicting findings.

Furthermore, it should be noted that EEG measures reconstructed from sparsely sampled sensor signals on the scalp into the three-dimensional brain space can result in source activity seeping into adjacent regions, which can distort connectivity measures (Palva et al. 2018). In contrast, the more accurate source identification of the brain connectome using fMRI has provided useful and meaningful information to explain a wide range of pathological conditions, including OCD (Friston 2002; Gürsel et al. 2018; Iwabuchi et al. 2015).

### Resting-state fMRI connectivity

#### The default mode network

Our review acknowledges the conflict between findings of both higher and lower resting-state FC within the DMN in OCD, highlighting the complexity of neural activity patterns in this network. However, despite contradictory findings (i.e. higher FC in some studies and lower FC in others), it is worth noting that studies revealing lower DMN FC in OCD commonly localized this to anterior regions (Beucke et al. 2014; Fan et al. 2023; Jang et al. 2010), while those indicating higher DMN FC in OCD often pinpoint it to posterior regions (Coutinho et al. 2016; Fan et al. 2017a). This observation suggests that OCD may affect DMN FC differently depending on the brain regions within the DMN. This perspective aligns with recent research challenging the notion of the DMN as a uniform and cohesive system, suggesting a developing understanding of its functional organization in OCD.

Decreased resting-state FC within the DMN in OCD groups compared to HC was reported in a large number of studies (10 in 25 studies that examined the DMN) (Beucke et al. 2014; Cui et al. 2020; Fan et al. 2023; Jang et al. 2010; Ma et al. 2022; Peng et al. 2014a; Peng et al. 2014b; Shan et al. 2019; Wang et al. 2019; Xu et al. 2023a). Additionally, one EEG study (Choi et al. 2021) also reported decreased beta-band FC within the DMN. The convergence of these results across EEG and fMRI modalities underscores the robustness of DMN connectivity alterations in OCD. The DMN is activated when individuals engage in internally focused thoughts, such as self-referential thoughts, autobiographical memory retrieval or envisioning the future (Gusnard et al. 2001). Therefore, the DMN is believed to play a crucial role in supporting adaptable mental simulations related to oneself and aiding in the retrieval of episodic memories, which serves the purpose of utilizing past experiences, and anticipating and assessing future events (Buckner et al. 2008). Individuals with OCD are known to be preoccupied with intrusive and persistent obsessions that are linked to negative external stimuli. Cognitive inflexibility arising from rigid conceptual frameworks has also been recognized as a cognitive trait characteristic of OCD (Gu et al. 2008). Therefore, it can be conceptualized that impaired DMN connectivity might render flexible mental simulations difficult for OCD patients.

It is noteworthy that several studies have localized the decreased FC to the anterior regions of the DMN, such as the mPFC (Beucke et al. 2014; Fan et al. 2023; Jang et al. 2010). From a clinical standpoint, OCD often involves abnormal cognitive processing related to the self. Obsessions, in particular, are typically experienced as ego-dystonic, meaning their content contradicts the patient's self-perception (Purdon and Clark 1999). Given that the mPFC is implicated in the mental representation of the self and the certainty of self-view (D’Argembeau et al. 2012), reduced connectivity in this brain region may hinder the ability to dissociate from obsessional content. This impaired recruitment of the mPFC self-system could potentially contribute to the occurrence of obsessive, ego-dystonic thoughts.

Furthermore, another study that reported decreased resting-state FC within the DMN (Peng et al. 2014b), demonstrated that unaffected siblings of OCD participants also exhibited impairments in FC within the PCC, a vital node of the DMN. Furthermore, impaired cognitive flexibility and motor inhibition were found in relatives of OCD patients (Chamberlain et al. 2007), both traits that are known to be linked to the PCC (Pearson et al. 2011). However, further research is essential to clarify the specific FC findings within the PCC in first-degree relatives, potentially providing brain-based markers for OCD and aiding in the identification of risk genes.

Intriguingly, a large body of literature (15 in 25 studies that examined the DMN) has also reported increased intra-network FC within the DMN (Coutinho et al. 2016; Fan et al. 2017a; Göttlich et al. 2015; Hou et al. 2013; Hou et al. 2014; Kinay et al. 2021; Koçak et al. 2012; Luo et al. 2021; Park et al. 2022; Shi et al. 2021; Takagi et al. 2017; Tang et al. 2023; Tikoo et al. 2020; Weber et al. 2014; Xu et al. 2023b). Recent imaging studies reveal a nuanced perspective on the DMN, challenging the notion of it being a uniform system. Instead, findings suggest a dissociation within the DMN, where the anterior component exhibits heightened activity during self-referential and emotional tasks, while the posterior regions become more prominent during tasks related to episodic memory and perceptual processing (Coutinho et al. 2016; Zhu et al. 2012). Several studies have reported the increase in intra-network FC to be localized to the posterior DMN regions, such as the precuneus (Coutinho et al. 2016) and superior parietal gyrus (Fan et al. 2017a). Therefore, abnormally high posterior DMN FC may be functionally linked to deficits in episodic memory that are present in OCD patients. Episodic memory is crucial for everyday belief updating (Davies and Coltheart 2000), and individuals with OCD often harbor irrational beliefs which can underpin their obsessive-compulsive symptoms. The observed deficits in episodic memory may align with the clinical observation that OCD patients struggle to form or utilize episodic memories to correct their obsessive-compulsive beliefs.

#### Cortico-striato-thalamo-cortical circuits

Altered FC within the sensorimotor and limbic sub-circuits were found to be the most consistently reported findings within the CSTC loops. The sensorimotor CSTC circuit connects the premotor cortex, putamen and thalamus and mediates transitions from goal-directed behaviors to habitual behaviors and automatic responses (Boedhoe and van den Heuvel 2018; van den Heuvel et al. 2016). Several studies have documented decreased connectivity within the Sensorimotor CSTC in OCD groups compared to HC (Cui et al. 2020; Deng et al. 2019; Lv et al. 2022; Moreira et al. 2019; Sha et al. 2020b; Xu and Zhang 2023; Yang et al. 2019). It is known that individuals with OCD have impaired sensorimotor functions such as sensory gating (Ahmari et al. 2012; Rossi et al. 2005), indicating the potential relevance of this circuit in the pathophysiology of OCD. Conversely, several studies have also reported significantly higher FC within the sensorimotor CSTC circuitry in OCD groups compared to HC (Armstrong et al. 2016; Cano et al. 2018; Chen et al. 2019; Hou et al. 2013; Kim et al. 2019; Park et al. 2020; Tikoo et al. 2020; Xu et al. 2021; Ye et al. 2020; Zhao et al. 2021). Moreover, the postcentral gyrus and supramarginal gyrus, which are both vital nodes of the sensorimotor CSTC circuit, have been found to show greater gray matter volume, metabolic rates and gyrification in OCD patients compared to HC (Subirà et al. 2015; Tang et al. 2016). However, one study reporting raised sensorimotor CSTC connectivity found the increased FC to be negatively correlated to OCD clinical symptom severity (Park et al. 2020). Therefore, it may be that the increased connectivity between sensorimotor CSTC regions reflects a compensatory mechanism for obsessive-compulsive symptoms rather than contributing towards the pathogenesis of OCD.

The limbic system includes subcortical structures such as the amygdala, as well as cortical structures such as the vmPFC, and is thought to play a major role in the regulation of emotion, memory and spatial orientation (Catani et al. 2013). Decreased FC within the limbic CSTC circuitry was identified in several studies (Anticevic et al. 2014; Cyr et al. 2021; Fullana et al. 2017; Göttlich et al. 2014; Haynes et al. 2018; Posner et al. 2014; Zhao et al. 2021). Functional integration within this limbic CSTC network is thought to mediate reinforcement learning (Bogacz and Larsen 2011; Costa 2007) and behavioral selection via connections to the basal ganglia (Graybiel 1998), the disturbance of which could explain why OCD patients choose inappropriate actions for specific circumstances, as a result of an inability to use new conditions as cues to update behavior (Figee et al. 2011), leading to the repetition of compulsions and cognitive rigidity (Bradbury et al. 2011). However, contradictory findings of enhanced connectivity within the limbic CSTC have also been reported (Apergis-Schoute et al. 2018; Calzà et al. 2019; de Vries et al. 2019; Kim et al. 2019; Yang et al. 2019). Hou et al. (2014) suggested that increased limbic FC may be an endophenotype for OCD as the finding was not correlated with disease severity and both patients and unaffected relatives showed similar differences compared to HC (Hou et al. 2014). Furthermore, the cortical structures of the limbic system (vmPFC) are thought to play a role in the regulation of emotions through implicit inhibitory control over the amygdala (Etkin et al. 2011). Therefore, increased connectivity between the cortical and subcortical limbic structures may imply an increased implicit effort to regulate emotions at rest, reflecting a compensatory mechanism rather than a pathophysiological marker (de Vries et al. 2019).

#### Inter-network connectivity and the “triple network model”

While traditional perspectives have associated alterations within individual brain networks with the pathophysiology of OCD (Vythilingum and Stein 2003), recent insights emphasize the inadequacy of these models in accounting for the complex interactions with other brain regions (Reess et al. 2016). In this context, the most consistently reported inter-network connectivity finding in our review was decreased FC between the DMN and SN (Chen et al. 2018; Geffen et al. 2022; Luo et al. 2021; Shi et al. 2021; Versace et al. 2019; Zhang et al. 2011; Zhou et al. 2022), both of which are vital networks within the TNM. The TNM encompasses the FPN (linked to external processes and goal-driven actions), DMN (associated with internal processes and self-referential thoughts) and SN (involved in switching between internal attention and goal-oriented behavior). In this conceptual framework, the SN functions as an intermediary between the FPN and the DMN, both of which exhibit an anticorrelated relationship; when one network is active, the other undergoes suppression. Poor connectivity between the DMN and SN may be associated with OCD patients’ difficulty in disengaging from internal self-referential thoughts to adapt to the changing external environment, which could present as both cognitive and behavioral disturbances simultaneously (Fan et al. 2017a). Additionally, reduced SN-DMN connectivity may contribute towards decreased sustained attention (Posner et al. 2017) and poor insight (Fan et al. 2017b) in individuals with OCD. Furthermore, several studies have reported hyperconnectivity between the SN and FPN in the OCD group compared to HC (Fan et al. 2017a; Li et al. 2012; Xu et al. 2023a; Yun et al. 2017; Zhou et al. 2022). This could be related to the known maladaptive cognitive performance in OCD patients, including intractable preoccupations and failure to flexibly adapt towards increasing cognitive load during working memory or executive planning tasks (Liang et al. 2016; Van Den Heuvel et al. 2005). Together, these findings may suggest a connectivity bias within the SN, leading to reduced regulation of DMN activity, and possibly increased engagement of the FPN in processing cognitions that are typically driven by DMN activity.

### Task-related fMRI connectivity

Although both resting-state DMN hyper- and hypoconnectivity have been reported, only one report of task-related DMN hyperconnectivity was found during a monetary reward task (Koch et al. 2018). The DMN is active during rest and deactivated during cognitive task performance (Gusnard et al. 2001). Therefore, the reported hyperconnectivity could be a result of failure to deactivate the DMN in OCD. A similar phenomenon has also been reported in other mental health conditions such as schizophrenia (Pomarol-Clotet et al. 2008) and autism (Spencer et al. 2012). Alternatively, the DMN hyperconnectivity may stem from a failure of regulation by the SN within the TNM framework. Another study reported significantly higher connectivity within the SN during a multisource inference task (Cocchi et al. 2012). It is known that the SN represents major nodes of a central autonomic network supporting autonomic arousal and interoceptive awareness (Craig 2009). OCD patients may experience heightened autonomic arousal during the demanding rest-to-task transition periods, leading to hyperconnectivity within the SN.

In the context of the task-related findings within the TNM, contrasting observations were noted compared to resting-state findings. One study reported SN-DMN hyperconnectivity during error trials of an anti-saccade task (Agam et al. 2014). This finding suggests that individuals with OCD struggle to disengage from self-evaluative processes following errors, impeding their ability to redirect attention effectively to the task-at-hand (Stern et al. 2011). Furthermore, another study reported SN-FPN hypoconnectivity during a thought action fusion task (Lee et al. 2022). This further substantiates the hypothesis that cognitive inflexibility in OCD patients leads to poor engagement of the task-positive FPN and impaired disengagement of the task-negative DMN during cognitive tasks (Gürsel et al. 2018).

In the task-related within network connectivity findings, the most consistently reported observation was increased FC within the FPN. Three studies reported significantly increased FC within the FPN in the OCD group compared to HC during a set shifting task (Kim et al. 2022), a cued task switching paradigm (Liu et al. 2023) and a cognitive reappraisal task (Picó-Pérez et al. 2022). Task switching and set shifting are both executive functions that involve the ability to shift attention between one task and another, and are thought to be subcategories of the broader concept of “cognitive flexibility” (Jersild 1927). Similarly, the cognitive reappraisal task engages selective attention and cognitive control, serving to guide focus towards relevant stimulus features. The cognitive reappraisal task also involves the retention of reappraisal goals and the content of one's reinterpretation within the realm of conscious thought (Ochsner and Gross 2014). Therefore, FPN hyperconnectivity may be an exaggerated response to the cognitive demand during these tasks, potentially stemming from the inherent cognitive inflexibility in OCD patients. Furthermore, given that OCD participants typically find these tasks more challenging than HC (Picó-Pérez et al. 2022), an increase in their network activation may occur as a compensatory mechanism to facilitate task performance.

### Limitations and future directions

Both EEG and fMRI have strengths and limitations when measuring brain FC. While EEG excels in temporal resolution, providing millisecond-level timing precision, it has lower spatial resolution and therefore, lacks precision in pinpointing the exact location of neural activity (Song et al. 2015). fMRI has lower temporal resolution as it measures changes in blood flow, which are relatively slow compared to electrical activity recorded by EEG. However, fMRI captures deeper brain structures with high accuracy (Menon and Goodyear 2001). Our review has identified several contradictory findings, which may be related to these respective limitations. Additionally, while our review predominantly includes fMRI studies, reflecting the current research landscape, the inclusion of EEG studies, although fewer in number, adds valuable insights into the temporal dynamics of brain activity in OCD. This disparity highlights the need for more future research using EEG to assess FC in OCD, and to adopt multimodal neuroimaging approaches, combining the strengths of both fMRI and EEG. Future studies could consider combining data from both modalities, or using a technique that combines the benefits of both, such as functional near infrared spectroscopy (fNIRS), magnetoencephalography (MEG), or simultaneous fMRI-EEG, potentially offering a more comprehensive understanding of the underlying FC findings in OCD. One of the significant challenges encountered in our review was the variability in network definitions across different studies, which could potentially impact the generalisability of our findings. For instance, some studies used different criteria or parcellation schemes to define networks, leading to variations in which brain regions are included within each network. To address this issue, future research should standardize network definitions or report the implementation of both methods as per recent multiverse analysis approaches (Steegen et al. 2016), which would enhance comparability and enable more robust identification of altered FC patterns.

A further notable challenge in synthesizing findings from both EEG and fMRI studies is the variability in preprocessing approaches. For example, in fMRI preprocessing, the use of global signal regression can introduce negative correlations in resting-state FC analysis, affecting the detection of overall FC patterns (Murphy et al. 2009a). In our review, the substantial heterogeneity in preprocessing pipelines across studies made it infeasible to conduct detailed subgroup analyses to evaluate the specific impact of each preprocessing method on the overall findings. To enable future research to address this issue, we emphasize the importance of standardizing preprocessing approaches to enhance the comparability of results across studies, facilitate meta-analyses and improve the reliability of conclusions drawn from the data.

The analysis of connectivity measures can be approached through various techniques. In the EEG studies under review and those conducted previously (Miljevic et al. 2023), diverse analysis methods have been employed, posing challenges in comparing results and drawing accurate conclusions. It is worth noting that a recently published guide and checklist offer standardization for EEG connectivity analyses (Miljevic et al. 2021). Given that the majority of EEG studies included in this review scored poorly on this checklist (Supplementary Table S3), caution is warranted in interpreting their results. Furthermore, encouraging future studies to adhere to this guideline and employ optimal methods would enhance the consistency of results and facilitate meaningful comparisons across studies. Additionally, establishing a similar guideline for fMRI connectivity analysis with optimal techniques would be beneficial. A further limitation of our review is the variability in the definitions of frequency bands across the included EEG studies. This lack of standardization in frequency band definitions could potentially impact the comparability of results across studies.

A notable limitation of our review is the reliance on a qualitative summary of disrupted brain connectivity in OCD, without the use of quantitative methods (i.e. coordinate-based meta-analysis). Previous research has demonstrated the utility of such quantitative methods in evaluating disrupted functional connectivity networks across various psychiatric disorders (Sha et al. 2019). The variability in network definitions and seed points used across studies introduces significant heterogeneity, which we aimed to address by mapping reported brain regions to widely accepted functional networks. However, future research would benefit from standardizing network definitions and integrating coordinate-based methods to synthesize findings more precisely and elucidate potential brain connectivity mechanisms underlying OCD.

This review also included multiple studies examining FC before and after the application of neuromodulation methods, such as DBS (Figee et al. 2013) and behavioral therapies (Cyr et al. 2021; Fullana et al. 2017; Gao et al. 2021). Furthermore, there are several studies that investigated the effects of targeted TMS therapy on brain network connectivity (Cocchi et al. 2018; Mantovani et al. 2021). While not the main focus of this article, pre- to post-treatment FC changes and their relationship to treatment response could offer valuable insights into OCD mechanisms and potential therapies. For example, in our recent pilot study, individualized tACS demonstrated significant improvement in OCD symptoms (Perera et al. 2023a), adding to the growing body of research highlighting the promising outcomes of tACS therapy in OCD treatment (Grover et al. 2021; Klimke et al. 2016). This underscores the potential for future studies to explore alterations in brain FC associated with tACS therapy, providing valuable insights into its underlying mechanisms in OCD.

Our review focused on fMRI and EEG studies due to their prevalence and established protocols in OCD research. This focus allowed for a comprehensive and coherent synthesis of findings. However, we acknowledge the value of other neuroimaging techniques such as fNIRS and MEG, which offer unique insights into brain function. Future reviews could benefit from including these modalities to provide a more holistic understanding of OCD. Additionally, our literature search uncovered no task-related EEG studies that reported FC findings in OCD to date. Task-related EEG studies can provide important insights into neural mechanisms underlying OCD. Therefore, future research should investigate FC differences using task-related EEG data to enhance our understanding of the disorder's neural underpinnings.

## Conclusions

OCD is a chronic condition significantly impacting the quality of life of patients, yet successful treatment remains challenging due to our limited understanding of its underlying pathophysiology. Neuroimaging studies, utilizing techniques such as EEG and fMRI, have examined brain FC in OCD. Our review considered 166 studies (10 EEG and 156 fMRI). In EEG studies, OCD exhibited lower delta and alpha FC, with inconsistent findings in other frequency bands. Resting-state fMRI studies, however, presented conflicting reports of both increased and decreased FC within the DMN and the sensorimotor CSTC circuit. Notably, decreased connectivity between the DMN and SN at rest suggests a potential link to the TNM, implicated in OCD pathophysiology. Task-related hyperconnectivity between the DMN-SN and hypoconnectivity between the SN-FPN point towards cognitive inflexibility in OCD, potentially rooted in TNM dysfunction. In conclusion, our neuroimaging review unveils a complex FC landscape in OCD, highlighting complex interplays within and between brain networks. However, the presence of conflicting findings underscores the necessity for targeted research using standardized methods to deepen our understanding of the underlying pathophysiology of OCD.

## Supplementary Material

Supplementary_Material_V3_bhae327
