# Supplementary material for: Exploring functional connectivity in large-scale brain networks in obsessive-compulsive disorder: a systematic review of EEG and fMRI studies
Source: Cereb Cortex. 2024 Aug 16;34(8):bhae327. doi: 10.1093/cercor/bhae327 (PMC11329673; doi:10.1093/cercor/bhae327)
Supplement: Supplementary_Material_V3_bhae327 [file supplementary_material_v3_bhae327.pdf]

Supplementary Material

Supplementary Table S1 – Large-scale brain networks and involved brain regions

Brain Network	Brain Regions
Default Mode Network (DMN)	Medial prefrontal cortex (mPFC), posterior cingulate cortex (PCC), inferior parietal cortex, precuneus, angular gyrus, lingual gyrus, middle/superior temporal gyrus, parahippocampal gyrus
Limbic cortico-striato-thalamo-cortical (CSTC) circuit	Ventromedial prefrontal cortex (vmPFC), amygdala, hippocampus, thalamus
Dorsal cognitive CSTC circuit	Dorsolateral prefrontal cortex (DLPFC), caudate nucleus, thalamus
Sensorimotor CSTC circuit	Supplementary motor area (SMA), premotor cortex, putamen, thalamus, precentral gyrus
Ventral motivational CSTC circuit	Orbitofrontal cortex (OFC), Nucleus accumbens (NAc), thalamus
Frontoparietal Network (FPN)	DLPFC, inferior and superior parietal lobule, thalamus, middle cingulate gyrus (midcingulate cortex), dorsal precuneus, head of caudate, superior & inferior and middle frontal gyrus, middle occipital gyrus, dorsal anterior cingulate cortex (dACC)
Salience Network (SN)	Anterior insula, anterior cingulate cortex (ACC), operculum
Visual Network (VN)	Primary visual cortex, occipital lobe, occipital pole, lateral geniculate nucleus

Supplementary Table S2 – Resting-state fMRI connectivity within and between large-scale brain networks

Each cell represents the cumulative number of OCD participants supporting increased (Table S2a) or decreased (Table S2b) functional connectivity between the large-scale brain networks mentioned in the corresponding column and row names. The diagonal represents the within network connectivity findings.

	**DMN**	**CSTC (limbic)**	**CSTC (VMC)**	**CSTC (SM)**	**CSTC (DC)**	**FPN**	**SN**	**Cerebellum**	**VN**
**DMN**	528	NA	NA	NA	NA	NA	NA	NA	NA
**CSTC (limbic)**	92	291	NA	NA	NA	NA	NA	NA	NA
**CSTC (VMC)**	61	56	325	NA	NA	NA	NA	NA	NA
**CSTC (SM)**	64	92	23	458	NA	NA	NA	NA	NA
**CSTC (DC)**	63	29	140	88	252	NA	NA	NA	NA

**FPN**	129	133	74	71	63	357	NA	NA	NA
**SN**	224	0	70	172	128	264	245	NA	NA
**Cerebellum**	269	38	0	111	108	173	0	47	NA
**VN**	0	45	0	0	0	0	0	20	40

Table S2a - Increased functional connectivity

	**DMN**	**CSTC (limbic)**	**CSTC (VMC)**	**CSTC (SM)**	**CSTC (DC)**	**FPN**	**SN**	**Cerebellum**	**VN**
**DMN**	525	NA	NA	NA	NA	NA	NA	NA	NA
**CSTC (limbic)**	17	253	NA	NA	NA	NA	NA	NA	NA
**CSTC (VMC)**	58	88	182	NA	NA	NA	NA	NA	NA
**CSTC (SM)**	248	159	256	381	NA	NA	NA	NA	NA
**CSTC (DC)**	90	44	149	176	111	NA	NA	NA	NA
**FPN**	60	37	128	126	85	297	NA	NA	NA
**SN**	285	169	80	211	47	201	213	NA	NA
**Cerebellum**	77	64	0	105	0	171	0	229	NA
**VN**	24	0	0	223	30	25	24	61	154

Table S2b - Decreased functional connectivity

Supplementary Table S3 – Study quality of EEG connectivity studies included in the review

Study quality was calculated using the guide and checklist presented by Miljevic et al. (2021). Quality levels are categorised as low (score 0-3), moderate (score 3.5-4.5) and high (score ≥5).

**Question no.**	Perera et al. (2023)	Ozcoban et al. (2018)	Desarkar et al. (2007)	Choi et al. (2021)	Velikova et al. (2010)	Tan et al. (2022)	Tan et al. (2019)	Olbrich et al. (2013)	Yazdi-Ravandi et al. (2018)	Saifutdinova et al. (2016)
**1**	1	0	0	0.5	0	0	0	0.5	0.5	0
**2**	1	0	0	0	0	0.5	0	0	0	0
**3**	0	1	0.5	0	0.5	0	0	1	0	0
**4**	1	0.5	0.5	0.5	1	1	0.5	1	0.5	1
**5**	0	0	0	1	0.5	0.5	0	1	0.5	1
**6a**	-	-	0	-	1	-	-	1	1	-
**6b**	1	0.5	-	0.5	-	0.5	1	-	-	1
**7**	1	0	0	0	0	0	0	0	0	0
**Total**	**5**	**2**	**1**	**2.5**	**3**	**2.5**	**1.5**	**4.5**	**2.5**	**3**

Table S3a - Study quality scores of EEG connectivity studies included in this review

	0	0.5	1
1. Re-referencing technique:	Single, mastoid / ear, nose, or not presented ☐	CAR ☐	REST, rCAR, or Laplacian ☐
2. Epoch length:	<3 s ☐	4-6 s ☐	>6 ☐
3. Number of sample epochs:	<50 ☐	50-100 ☐	>100 ☐
4. Artefact rejection technique (for specifics see "Checklist Scoring Specifics" section):	None ☐	Noisy epochs, or channels rejected ☐	All types of artefacts addressed ☐
5. Control for volume conduction (for specifics see "Checklist Scoring Specifics" section):	None ☐	Lag, weighted, source, or Laplacian ☐	(Lag or weighted) & (source-space or Laplacian) ☐
6a. Control for multiple comparisons (for specifics see "Checklist Scoring Specifics" section):	No post hoc ☐	Invalid post hoc control or p-value = 0.01 ☐	Valid post hoc control (If network / cluster - based stats use 6b instead) ☐
6b. Only for studies assessing network metrics:		Arbitrary thresholding / not model-driven ☐	Model or data - driven: threshold or weighted ☐
7. Sample size estimation and consideration:	No considerations ☐	Some consideration (i.e., *N* obtained from published literature) ☐	Statistical consideration (i.e., a priori *N* calculation) ☐
**Scores:**	________	________	________
**Total score & QR:**	__________		

Note. 0 = not recommended for use; 0.5 = not optimal; and 1 = optimal for use.

Table S3b - EEG connectivity study quality checklist

Author (year)	OCD sample (Male, Female, Mean age, SD)	Comparison sample (Male, Female, Mean age, SD)	Key findings	Involved neurocircuitry and direction of connectivity findings	Functional Connectivity Analysis Method
(Abe et al. 2015)	37 (15M, 22F; 30.4±7.5y)	38 (18M, 20F; 32.7±9.7y)	Increased FC between OFC and ventral striatum (NAc) in OCD.	CSTC (VMC) ↑	ROI-based voxel-wise analysis
(Anticevic et al. 2014)	27 (15M, 12F; 36.37±13.6y)	66 (41M, 25F; 33±10.4y)	Clusters of decreased FC within L-lateral PFC. Increased FC between R-putamen and L-cerebellum, NAc and ACC.	CSTC (limbic) ↓<br>CSTC (SM) – cerebellum ↑<br>CSTC (VMC) – SN ↑	ROI-based voxel-wise analysis
(Apergis-Schoute et al. 2018)	38 (21M, 17F; 40.7±13.1y)	34 (18M, 16F; 38.3±13.3y)	Increased FC from vmPFC to temporal and occipital lobes, cerebellum and the motor cortex	CSTC (limbic) ↑<br>CSTC (limbic) – cerebellum ↑	ROI-based voxel-wise analysis
(Armstrong et al. 2016)	21 (13M, 8F; 12.7±2.8y)	20 (11M, 9F; 13.4±1.8y)	Less efficient global network connectivity in OCD. Higher internal FC in sensorimotor, supplementary motor and frontal polar cortex.	CSTC (SM) ↑	Graph theory-based approach
(Becker et al. 2023)	23 (0M, 23F; 14±3.84y)	44 (0M, 44F; 14.55±3.88y)	Significantly higher FC within the SN and between the SN and CSTC-VMC in OCD.	SN ↑<br>SN – CSTC (VMC) ↑	Parcellation-based network analysis
(Bernstein et al. 2016)	15 (8M, 7F; 15.3±2.1y)	13 (7M, 6F; 16±1.8y)	Decreased FC between L-putamen and OFC, IFG, insula and operculum in OCD.	CSTC (SM) – CSTC (VMC) ↓<br>CSTC (SM) – SN ↓	ROI-based voxel-wise analysis
(Beucke et al. 2013)	23 (11M, 12F; 29.1±9.1y)	23 (11M, 12F; 28.7±8.9y)	Increased FC between the OFC and subthalamic nucleus, putamen in OCD.	CSTC (VMC) ↑<br>CSTC (VMC) – CSTC (SM) ↑	ROI-based voxel-wise analysis
(Beucke et al. 2014)	46 (20M, 26F; 30.7±9.4y)	46 (20M, 26F; 30.3±8.8y)	Significantly reduced connectivity within the dorsal medial prefrontal cortex subsystem of DMN (PCC, dmPFC).	DMN ↓	ROI-based voxel-wise analysis
(Calzà et al. 2019)	44 (14M, 30F; 33.32±11.4y)	40 (19M, 21F; 34.12±8.8y)	Increased FC between several basal ganglia (Subthalamic nucleus, globus pallidus) in OCD.	CSTC (VMC) ↑<br>CSTC (limbic) ↑	ROI-based voxel-wise analysis
(Cano et al. 2018)	86 (43M, 43F; 34.38±9.4y)	104 (59M, 45F; 34.18±10.4y)	Increased FC between STN and pre-motor cortex, decreased FC between STN and lenticular nuclei in OCD.	CSTC (SM) ↑<br>CSTC (SM) – CSTC (limbic) ↓	ROI-based voxel-wise analysis
(Cao et al. 2022a)	88 (56M, 32F; 27.41±6.6y)	88 (56M, 32F; 25.95±7.8y)	Early onset group: increased amygdala-precuneus and decreased amygdala-OFC FC. Late onset: increased amygdala-PCG.	CSTC (limbic) – FPN ↑<br>CSTC (limbic) – CSTC (VMC) ↓	ROI-based voxel-wise analysis
(Cao et al. 2022b)	92 (57M, 35F; 29.42±8.7y)	90 (55M, 35F; 28.34±10.9y)	Decreased FC between amygdala and L-insula. Increased FC between amygdala and SMA, PCG, and superior temporal gyrus.	CSTC (limbic) – SN ↓<br>CSTC (limbic) – CSTC (SM) ↑<br>CSTC (limbic) – DMN ↑	Parcellation-based network analysis
(Chen et al. 2016a)	30 (24M, 6F; 26.23±5.7y)	30 (23M, 7F; 28.17±7.7y)	Decreased FC within the dorsal cognitive CSTC (caudate, thalamus) and increased FC between caudate and SMA, PCG	CSTC (DC) ↓<br>CSTC (DC) – CSTC (SM) ↑	ROI-based voxel-wise analysis
(Chen et al. 2016b)	30 (24M, 6F; 26.23±5.7y)	30 (23M, 7F; 28.17±7.7y)	Increased FC within the FPN in OCD compared to HC.	FPN ↑	Parcellation-based network analysis, ROI-based voxel-wise analysis
(Chen et al. 2018)	40 (27M, 13F; 27.28±8.2y)	40 (27M, 13F; 27±8.3y)	Decreased intrinsic connectivity within SN, and decreased inter-network connectivity between SN and DMN, and FPN.	SN ↓<br>SN – DMN ↓<br>SN – FPN ↓	ROI-based voxel-wise analysis

(Chen et al. 2019)	23 (15M, 8F; 32.1±10.5y)	23 (15M, 8F; 31.4±10y)	Increased FC from thalamus to dACC, L-SMA and decreased FC to R-middle occipital gyrus.	CSTC (SM) ↑<br>CSTC (SM) – SN ↑<br>CSTC (SM) – VN ↓	ROI-based voxel-wise analysis
(Chen et al. 2021)	40 (27M, 13F; 27.28±8.2y)	38 (25M, 13F; 27.18±8.3y)	Decreased FC between NAc and BL-OFC, mPFC in OCD.	CSTC (VMC) ↓<br>CSTC (VMC) – DMN ↓	ROI-based voxel-wise analysis
(Cheng et al. 2013)	23 (8M, 15F; 31±10.26y)	23 (8M, 15F; 31.65±8.9y)	Increased FC between ACC and SFG, midbrain and SMA, and between PCC and OFG, DLPFC in OCD	SN – CSTC (SM) ↑<br>DMN – CSTC (DC) ↑<br>DMN – FPN ↑	ROI-based voxel-wise analysis, parcellation-based network analysis
(Coutinho et al. 2016)	10 (5M, 5F; 40±9.4y)	10 (5M, 5F; 38±8.8y)	Increased FC within the DMN (PCC, precuneus, mPFC, BL-inferior parietal cortex) in OCPD compared to HC	DMN ↑	Parcellation-based network analysis
(Cui et al. 2020)	40 (27M, 13F; 27.28±8.2y)	38 (25M, 13F; 27.18±8.3y)	Decreased FC within the DMN (L-PCC/lingual gyrus) and SM network (PCG) and increased FC within the FPN (DLPFC).	DMN ↓<br>CSTC (SM) ↓<br>FPN ↑	ROI-based voxel-wise analysis
(Cyr et al. 2020)	25 (12M, 13F; 12.8±2.9y)	23 (12M, 11F; 11±3.3y)	Decreased FC between L-angular gyrus and middle frontal gyrus in OCD.	DMN – FPN ↓	Parcellation-based network analysis
(Cyr et al. 2021)	25 (12M, 13F; 12.8±2.9y)	23 (11M, 12F; 11±3.3y)	Decreased FC between R-amygdala and vmPFC in OCD.	CSTC (limbic) ↓	ROI-based voxel-wise analysis
(de Vries et al. 2019)	39 (18M, 21F; 38±9.7y)	36 (17M, 19F; 39.4±11.3y)	Significantly higher FC within the fronto-limbic network (vmPFC to basal ganglia) in OCD.	CSTC (limbic) ↑	ROI-based voxel-wise analysis
(Deng et al. 2019)	46 (26M, 20F; 30.39±10.7y)	46 (26M, 20F; 31.83±10.3y)	Decreased FC in lingual gyrus, PCG, putamen in OCD.	CSTC (SM) ↓<br>CSTC (SM) – DMN ↓	ROI-based voxel-wise analysis (voxel-mirrored homotopic connectivity)
(Dikmeer et al. 2021)	30 (13M, 17F; 32.4± 10y)	31 (13M, 18F; 32.3±8.5y)	Significantly reduced FC between caudate and middle temporal/middle occipital cortex.	CSTC (DC) – VN ↓<br>CSTC (DC) – DMN ↓	Parcellation-based network analysis
(Ding et al. 2023)	50 (29M, 21F; 26.36±8y)	50 (32M, 18F; 25.60±7.9y)	Decreased dynamic FC between L-superior temporal gyrus and cerebellum, and between R-SMA and R-DLPFC.	DMN – Cerebellum ↓<br>CSTC (SM) – FPN ↓	ROI-based voxel-wise analysis (dynamic FC)
(Dong et al. 2020)	35 (24M, 11F; 23.6±5.5y)	35 (18M, 17F; 27.8±6.7y)	Increased FC between L-caudate and OFC in OCD.	CSTC (DC) – CSTC (VMC) ↑	ROI-based voxel-wise analysis
(Fajnerova et al. 2020)	36 (18M, 18F; 33.26±8.2y)	36 (19M, 17F; 33.26±6.7y)	Increased FC between precuneus-angular gyrus and DLPFC. Decreased FC between caudate-thalamus and ACC-limbic lobe.	CSTC (DC) ↓<br>FPN – DMN ↑<br>SN – CSTC (limbic) ↓	ROI-based voxel-wise analysis
(Fan et al. 2017b)	40 (26M, 14F; 22.89±5.6y)	24 (9M, 15F; 21.92±2.2y)	Significantly increased FC within the SN (R-anterior insula, L-dACC) in OCD group with good insight. Decreased connectivity between R-AI and mOFC in poor insight group.	SN ↑<br>SN – CSTC (VMC) ↓	ROI-based voxel-wise analysis
(Fan et al. 2017a)	35 (19M, 16F; 24.23±5.6y)	32 (12M, 20F; 22.53±2.2y)	Increased FC within the DMN, FPN, SN and between SN-DMN and SN-FPN in OCD.	DMN ↑ FPN ↑<br>SN ↑<br>SN – DMN ↑<br>SN – FPN ↑	Parcellation-based network analysis
(Fan et al. 2018)	35 (19M, 16F; 23.86±5.5y)	36 (13M, 23F; 22.86±2.7y)	Significantly reduced FC between R-mPFC with SFG and BL-thalamus in OCD	DMN – FPN ↓	ROI-based voxel-wise analysis

(Fan et al. 2023)	165 (84M, 81F; 23.65±6.7y)	79 (35M, 44F; 23.78±5.4y)	Decreased FC within the response inhibition network involving medial prefrontal cortex and inferior parietal lobe.	DMN ↓	ROI-based voxel-wise analysis
(Fitzgerald et al. 2011)	60 (27M, 33F; 19.75±3.03y)	61 (28M, 33F; 19.83±3.48y)	Increased FC in dorsal cognitive CSTC (dorsal striatum, vmPFC) in all age groups. Youngest age group showed decreased FC in CSTC loops involved in cognitive control (dorsal striatum/thalamus, dACC).	CSTC (DC) ↑<br>CSTC (DC) – DMN ↓	ROI-based voxel-wise analysis
(Fullana et al. 2017)	73 (35M, 38F; 34.18±9.3y)	84 (41M, 43F; 33.68±9.8y)	Decreased FC between basolateral amygdala and vmPFC in OCD.	CSTC (limbic) ↓	ROI-based voxel-wise analysis
(Gao et al. 2019)	64 (36M, 28F; 29±6.9y)	60 (31M, 29F; 28.5±5.4y)	Significantly increased FC between the L-DLPFC and L-cerebellum in OCD.	CSTC (DC) – cerebellum ↑	ROI-based voxel-wise analysis
(Gao et al. 2021)	45 (25M, 20F; 28.7±6.7y)	40 (22M, 18F; 28.9±6.4y)	Increased FC between L-amygdala and R-middle frontal gyrus, amygdala and R-cuneus.	CSTC (limbic) – FPN ↑<br>CSTC (limbic) – VN ↑	ROI-based voxel-wise analysis
(Geffen et al. 2022)	24 (13M, 11F; 37.2±11.9y)	33 (15M, 18F; 35.7±11.5y)	Decreased FC between SN and DMN, between visual network and both DMN and SN in OCD.	SN – DMN ↓<br>VN – DMN ↓<br>VN – SN ↓	ROI-based voxel-wise analysis, parcellation-based network analysis
(Göttlich et al. 2014)	17 (5M, 12F; 30.4±9.6y)	19 (4M, 15F; 32.6±11.6y)	1. Decreased connectivity between the limbic CSTC to DMN, executive/attention network and basal ganglia in OCD.<br>2. Intra-network connectivity within the limbic network was decreased in OCD<br>3. Hyperconnectivity within FPN in OCD.	CSTC (limbic) – DMN ↓<br>CSTC (limbic) – FPN ↓<br>CSTC (limbic) ↓<br>FPN ↑	Graph theory-based approach
(Göttlich et al. 2015)	17 (5M, 12F; 32.6±11.6y)	19 (4M, 15F; 30.4±9.6y)	Increased FC in the middle temporal gyrus and decreased FC in amygdala, hippocampus and ventral striatum	DMN ↑<br>CSTC (VMC) ↓	Graph theory-based approach
(Guo et al. 2022)	37 (22M, 15F; 27.22±8.6y)	37 (20M, 17F; 24.16±4.3y)	Decreased FC between cerebellum and FPN, limbic and SM networks in OCD	Cerebellum – FPN ↓<br>Cerebellum – CSTC (limbic) ↓<br>Cerebellum – CSTC (SM) ↓	ROI-based voxel-wise analysis
(Gürsel et al. 2020)	49 (16M, 33F; 34.42±12.1y)	41 (19M, 22F; 35.07±10y)	Decreased FC between L and R-FPN and between the L-FPN and SN in OCD.	FPN ↓<br>FPN – SN ↓	Parcellation-based network analysis
(Han et al. 2023)	100 (53M, 47F; 22.93±9.3y)	106 (53M, 53F; 23.09±5.6y)	Decreased FC between DMN and CSTC (SM) in OCD.	DMN – CSTC (SM) ↓	Parcellation-based network analysis
(Harrison et al. 2009)	21 (10M, 11F; 8.52±5.9y)	21 (10M, 11F; 26.2±3.4y)	Significantly higher FC between the OFC and ventral caudate/NAc in OCD.	CSTC (VMC) ↑	ROI-based voxel-wise analysis
(Harrison et al. 2013)	74 (42M, 32F; 33.1±8.3y)	74 (42M, 32F; 32.7±10.3y)	Increased FC between ventral caudate and OFC. Decreased FC between ventral caudate and BL insular cortex.	FPN – CSTC (VMC) ↑<br>FPN – SN ↓	ROI-based voxel-wise analysis
(Haynes et al. 2018)	37 (16M, 21F; 37.54±9.9y)	37 (16M, 21F; 34.03±11.3y)	Decreased FC within the CSTC (limbic) in OCD.	CSTC (limbic) ↓	ROI-based voxel-wise analysis
(Hong et al. 2018)	15 (7M, 8F; 24.4±5.4y)	15 (7M, 8F; 22.5±2.1y)	At baseline, increased FC between dACC-cingulate gyrus and decreased FC between dACC-superior frontal gyrus.	SN – DMN ↑<br>SN – FPN ↓	ROI-based voxel-wise analysis
(Hou et al. 2013)	33 (18M, 15F; 25.3±9.6y)	33 (18M, 15F; 25±9.1y)	Significantly increased FC within the CSTC circuit (BL-OFC, ACC, caudate, putamen, thalamus, L-inferior frontal gyrus) and DMN (PCC). Increased FC in CSTC correlates with OCD severity.	CSTC (VMC) ↑<br>CSTC (SM) ↑<br>DMN ↑	ROI-based voxel-wise analysis

(Hou et al. 2014)	39 (20M, 19F; 26.6±9.8y)	39 (20M, 19F; 26±6.3y)	Increased FC within caudate, OFC and middle temporal gyrus. Decreased FC within the VN and cerebellum in OCD compared to HC.	DMN ↑ CSTC (DC) ↑ CSTC (VMC) ↑ VN ↓ Cerebellum ↓	ROI-based voxel-wise analysis
(Jang et al. 2010)	22 (16M, 6F; 25.14±6.96y)	22 (16M, 6F; 24.36±4.02y)	Decreased FC within the DMN regions in OCD compared to HC, indicating fronto-subcortical dysfunction.	DMN ↓	ROI-based voxel-wise analysis
(Jia et al. 2020)	40 (27M, 13F; 27.28±8.2y)	38 (25M, 13F; 27.18±8.3y)	Decreased FC in OFC, thalamus, PCG, middle occipital gyrus in OCD.	CSTC (VMC) – CSTC (SM) ↓ CSTC (SM) – FPN ↓	ROI-based voxel-wise analysis
(Jung et al. 2017)	61 (36M, 25F; 25.64±6.5y)	61 (41M, 20F; 26.08±7.2y)	Decreased FC between OFC and dorsomedial striatum (dorsal caudate). Increased FC between ventral striatum (NAc) and mPFC.	CSTC (VMC) – CSTC (DC) ↓ CSTC (VMC) – DMN ↑	ROI-based voxel-wise analysis, parcellation-based network analysis
(Kang et al. 2013)	18 (12M, 6F; 24.9±5.9y)	18 (12M, 6F; 24.7±2.7y)	Increased FC between caudate and middle cingulate cortex and PCG in OCD	CSTC (DC) – FPN ↑	ROI-based voxel-wise analysis
(Kashyap et al. 2021)	20 (10M, 10F; 28.8±7y)	22 (10M, 12F; 28.18±6.7y)	Increased FC between cerebellar and VN nodes. Decreased FC between limbic CSTC and FPN.	Cerebellum – VN ↑ CSTC (limbic) – FPN ↓	Parcellation-based network analysis
(Kim et al. 2019)	102 (68M, 34F; 25.3±6.5y)	101 (62M, 39F; 25.4±6.9y)	Significantly larger FC within CSTC regions (temporal cortices, middle temporal gyrus, paracingulate gyrus, amygdala, hippocampus, putamen, thalamus)	CSTC (limbic) ↑ CSTC (SM) ↑	ROI-based voxel-wise analysis
(Kinay et al. 2021)	15 (5M, 10F; 15.27±1.5y)	15 (5M, 10F; 15.4±1.4y)	Increased FC within the anterior DMN and decreased FC within the cerebellum and FPN in OCD.	DMN ↑ Cerebellum ↓ FPN ↓	Parcellation-based network analysis
(Koçak et al. 2012)	12 (6M, 6F)	12 (6M, 6F)	Higher connectivity within DMN regions (BL-IPL, L-vmPFC) in OCD.	DMN ↑	ROI-based voxel-wise analysis
(Li et al. 2012)	20 (14M, 6F; 28.2±7y)	20 (14M, 6F; 28.2±7.3y)	Increased FC between R-anterior PFC and R-insula and middle cingulate cortex in OCD.	FPN ↑ FPN – SN ↑	ROI-based voxel-wise analysis
(Li et al. 2018)	20 (13M, 7F; 30.35±7.5y)	20 (13M, 7F; 30.55±7.8y)	Increased FC between the L-DLPFC and R-OFC in OCD.	CSTC (DC) – CSTC (VMC) ↑	ROI-based voxel-wise analysis
(Li et al. 2019)	45 (19M, 26F; 28.2±8.7y)	43 (20M, 23F; 28.3±8.3y)	Decreased FC between left thalamus-left orbital inferior frontal gyrus and R-thalamus-L inferior parietal gyrus in OCD.	CSTC (DC) – FPN ↓ CSTC (DC) ↓	ROI-based voxel-wise analysis
(Li et al. 2020a)	88 (56M, 32F; 29.16±8.7y)	88 (56M, 32F; 27.88±10.6y)	Decreased FC between the R-DLPFC and R-OFC in OCD.	CSTC (DC) – CSTC (VMC) ↓ FPN – CSTC (VMC) ↓	ROI-based voxel-wise analysis
(Li et al. 2020b)	42 (19M, 23F; 27.21±8.1y)	42 (19M, 23F; 28.31±8.4y)	Increased FC between L-ACC and R-middle temporal gyrus and between the middle temporal gyrus and cerebellum.	SN – DMN ↑ DMN – Cerebellum ↑	ROI-based voxel-wise analysis
(Liu et al. 2021)	50 (26M, 24F; 25.9±3.6y)	50 (25M, 25F; 23.7±2y)	Decreased FC between cerebellum and FPN in OCD.	Cerebellum – FPN ↓	Parcellation-based network analysis
(Luo et al. 2021)	29 (19M, 10F; 27.8±9.4y)	40 (25M, 15F; 27.9±9.2y)	Increased FC within DMN and SN, with negative coupling between DMN and SN in OCD.	DMN ↑ SN ↑ DMN – SN ↓	Parcellation-based network analysis
(Lv et al. 2020)	40 (27M, 13F; 27.28±8.2y)	38 (25M, 13F; 27.18±8.3y)	Increased FC between cerebellum and mPFC, middle temporal gyrus in OCD.	Cerebellum – DMN ↑	ROI-based voxel-wise analysis

(Lv et al. 2021)	40 (27M, 13F; 27.28±8.2y)	38 (25M, 13F; 27.18±8.3y)	Decreased FC in L-DLPFC, R-precuneus and L-PCG. Increased FC in L-thalamus and cerebellum.	CSTC (DC) – FPN ↓<br>CSTC (DC) – CSTC (SM) ↓<br>CSTC (SM) – Cerebellum ↑	ROI-based voxel-wise analysis
(Lv et al. 2022)	40 (27M, 13F; 27.28±8.2y)	38 (25M, 13F; 27.18±8.3y)	Decreased FC within the PCG and increased FC between R-thalamus, caudate and L-inferior parietal lobule and cerebellum.	CSTC (SM) ↓<br>CSTC (DC) – DMN ↑	ROI-based voxel-wise analysis
(Ma et al. 2022)	62 (31M, 31F; 28.83±7.4y)	60 (30M, 30F; 30.95±8.6y)	Decreased FC between the parahippocampal gyrus and PCG and superior temporal gyrus.	DMN ↓<br>DMN – CSTC (SM) ↓	ROI-based voxel-wise analysis
(Meunier et al. 2012)	18 (11M, 7F; 35.4±9.8y)	18 (15M, 3F; 32.7±6.9y)	Decreased FC in R-OFC and between OFC and PCC in OCD	CSTC (VMC) ↓<br>CSTC (VMC) – DMN ↓	Parcellation-based network analysis
(Moody et al. 2017)	43 (22M, 21F; 33±10.7y)	24 (14M, 10F; 31±12y)	No difference in FC between OCD and HC at baseline.	-	ROI-based voxel-wise analysis
(Moreira et al. 2017)	40 (13M, 27F; 26.28±6.6y)	40 (13M, 27F; 26.45±5.4y)	Decreased FC between OFC-ACC and lingual-PCG. Increased FC between thalamus-occipital lobe.	CSTC (VMC) – SN ↓<br>DMN – CSTC (SM) ↓<br>VN ↑	ROI-based voxel-wise analysis
(Moreira et al. 2019)	40 (13M, 27F; 26.52±6.6y)	40 (13M, 27F; 26.45±5.4y)	Reduced FC within and between visual and SM networks and increased FC between DMN-Cerebellum in OCD.	VN ↓<br>CSTC (SM) ↓<br>CSTC (SM) – VN ↓<br>DMN – Cerebellum ↑	Parcellation-based network analysis
(Murayama et al. 2021)	47 (18M, 29F; 33.3±11.9y)	62 (22M, 40F; 32.61±11y)	Significantly higher FC between the cerebellum (R-lobule VI) and L-precuneus	Cerebellum – DMN ↑	ROI-based voxel-wise analysis
(Nakamae et al. 2014)	20 (6M, 14F; 35.3±9.4y)	20 (9M, 11F; 32.9±6.9y)	Increased FC between the OFC and ventral striatum (NAc) in OCD	CSTC (VMC) ↑	ROI-based voxel-wise analysis, parcellation-based network analysis
(Naze et al. 2023)	52 (29M, 23F; 30.2±7.9y)	45 (27M, 18F; 32.5±8.7y)	Higher FC between the OFC and NAc, but lower FC between dorsal putamen and lateral-PFC	CSTC (VMC) ↑<br>CSTC (SM) – CSTC (DC) ↓	ROI-based voxel-wise analysis
(Park et al. 2020)	23 (19M, 4F; 27.74±5.4y)	23 (19M, 4F; 22.57±3.5y)	Increased FC between the putamen and several cortical regions (PCG, angular gyrus) in OCD.	CSTC (SM) ↑<br>CSTC (SM) – DMN ↑	ROI-based voxel-wise analysis
(Park et al. 2022)	107 (72M, 35F; 25.2±2.1y)	110 (69M, 41F; 25±4.9y)	Decreased FC within the CSTC (VMC) and increased FC within the DMN.	CSTC (VMC) ↓<br>DMN ↑	ROI-based voxel-wise analysis
(Peng et al. 2014b)	15 (10M, 5F; 26.7±4.8y)	28 (21M, 7F; 27.5±8.4y)	Significantly reduced FC within the DMN (PCC) and in OCD and increased FC with AI, R-inferior frontal lobe.	DMN ↓<br>DMN – SN ↑	ROI-based voxel-wise analysis
(Peng et al. 2014a)	30 (21M, 9F; 28±6.8y)	30 (22M, 8F; 27.3±8.2y)	Decreased FC within the DMN and increased FC between CSTC-SM and FPN.	DMN ↓<br>CSTC (SM) – FPN ↑	Graph theory-based approach
(Peng et al. 2022)	62 (45M, 17F; 26.8±8.3y)	73 (51M, 22F; 27.2±9.4y)	Significantly higher FC within the caudate (CSTC-DC) in OCD.	CSTC (DC) ↑	ROI-based voxel-wise analysis
(Picó-Pérez et al. 2019)	73 (43M, 30F; 37.74±10.19y)	42 (22M, 20F; 39.43±9.79y)	Reduced connectivity between R-amygdala and R-post central gyrus in OCD, significantly correlated to OCD severity.	CSTC (SM) – CSTC (limbic) ↓	ROI-based voxel-wise analysis
(Ping et al. 2013)	20 (16M, 4F; 27.1±8y)	20 (16M, 4F; 27.6±8.2y)	Increased FC between OFC and ventral ACC in OCD.	CSTC (VMC) – SN ↑	ROI-based voxel-wise analysis

(Posner et al. 2014)	23 (11M, 12F; 30.9±8.8y)	20 (11M, 9F; 32.6±10y)	Reduced FC within the limbic CSTC loop in unmedicated OCD compared to HC, positively correlated to OCD severity.	CSTC (limbic) ↓	ROI-based voxel-wise analysis
(Posner et al. 2017)	30 (16M, 14F; 29.1±7.9y)	32 (16M, 16F; 27.9±8y)	Significantly increased FC between vmPFC and AI in OCD.	DMN – SN ↑	ROI-based voxel-wise analysis
(Pujol et al. 2019)	160 (86M, 74F; 35.41±9.7y)	121 (66M, 55F; 34.6±10.2y)	Decreased FC between BL-SM cortex, BL-visual cortex, L-AI and BL-OFC in OCD.	CSTC (SM) – VN ↓<br>CSTC (SM) – SN ↓<br>CSTC (SM) – CSTC (VMC) ↓	ROI-based voxel-wise analysis
(Raposo-Lima et al. 2022)	75 (32M, 43F; 26±11y)	71 (24M, 47F; 25±6y)	Increased FC within the FPN and decreased FC within the visual network in OCD.	FPN ↑<br>VN ↓	Parcellation-based network analysis
(Reess et al. 2016)	41 (14M, 27F; 32.5±10y)	42 (18M, 24F; 31.8±8.3y)	Decreased FC between OFC-putamen, amygdala-AI in OCD.	CSTC (VMC) – CSTC (SM) ↓<br>CSTC (limbic) – SN ↓	Graph theory-based approach
(Sakai et al. 2011)	20 (8M, 12F; 30.9±9.3y)	23 (10M, 13F; 30.8±7.7y)	Increased FC between ventral striatum and OFC, DLPFC and vmPFC in OCD.	CSTC (VMC) ↑<br>CSTC (VMC) – CSTC (DC) ↑<br>CSTC (VMC) – CSTC (limbic) ↑	ROI-based voxel-wise analysis
(Sha et al. 2020b)	44 (15M, 29F; 23.61±4.8y)	43 (18M, 25F; 23.51±4.1y)	Significantly higher FC between L-caudate and BL-DLPFC in OCD.	CSTC (DC) ↑<br>FPN ↑	ROI-based voxel-wise analysis
(Sha et al. 2020a)	44 (15M, 29F; 23.61±4.8y)	43 (18M, 25F; 23.51±4.1y)	Decreased FC within the CSTC-SM and increased FC between CSTC-SM and cerebellum in OCD.	CSTC (SM) ↓<br>CSTC (SM) – Cerebellum ↑	Graph theory-based approach
(Shan et al. 2019)	20 (7M, 13F; 33.4±5.8y)	20 (9M, 11F; 35.2±5.3y)	Decreased FC in the L-middle temporal gyrus in OCD	DMN ↓	ROI-based voxel-wise analysis
(Shi et al. 2021)	41 (21M, 20F; 29.1±7.3y)	36 (18M, 18F; 29.7±7.5y)	Decreased FC between nodes of the SN and DMN with increased FC within nodes of the DMN	SN – DMN ↓<br>DMN ↑	Parcellation-based network analysis
(Shin et al. 2014)	25 (17M, 8F; 26.3±6.2y)	23 (13M, 10F; 26.9±5.5y)	Decreased FC within the FPN and between FPN and VN at baseline.	FPN ↓<br>FPN – VN ↓	Graph theory-based approach
(Stern et al. 2012)	30 (15M, 15F; 25.8±6.7y)	32 (15M, 17F; 28.35±8.5y)	Greater FC within the FPN and between FPN and DMN nodes (PCC, inferior parietal lobe, dmPFC) in OCD	FPN ↑<br>FPN – DMN ↑	ROI-based voxel-wise analysis
(Takagi et al. 2017)	56 (23M, 33F; 32.64±9.6y)	52 (26M, 26F; 29.4±7.5y)	Increased FC in OCD within FPN and DMN in OCD compared to HC.	FPN ↑<br>DMN ↑	Machine learning based approach
(Tang et al. 2023)	40 (25M, 15F; 29.7±9.4y)	57 (32M, 25F; 28.7±8.9y)	Increased FC between PCC-precuneus and decreased FC between frontal-middle cingulate gyrus.	DMN ↑<br>FPN ↓	ROI-based voxel-wise analysis
(Tian et al. 2016)	29 (21M, 8F; 26.6±8.1y)	29 (21M, 8F; 26.1±7.9y)	Increased FC in OCD than HC, distributed within the CSTC circuits (Brain hubs found at OFC, mPFC, DLPFC, ACC, PCC, insula). Significantly correlated with OCD symptom severity.	CSTC (VMC) – CSTC (DC) ↑<br>CSTC (DC) – CSTC (limbic) ↑<br>Cerebellum ↑	Graph theory-based approach
(Tikoo et al. 2020)	10 (7M, 3F; 10.9±2.5y)	11 (2M, 9F; 9.9±1.3y)	Increased FC in within nodes of CSTC (SM), DMN, FPN, SN in OCD.	CSTC (SM) ↑ DMN ↑<br>FPN ↑ SN ↑	Parcellation-based network analysis
(Tikoo et al. 2021)	11 (7M, 4F; 10.7±2.5y)	12 (3M, 9F; 10±1.2y)	Decreased FC between cerebellum (dentate nucleus) and L-PCG, L-inferior temporal gyrus and L-crus II in OCD.	Cerebellum ↓<br>Cerebellum – CSTC (SM) ↓<br>Cerebellum – FPN ↓	ROI-based voxel-wise analysis
(Tomiyama et al. 2019)	37 (16M, 21F; 33.49±11.4y)	40 (17M, 23F; 35.48±11.1y)	Increased FC between dorsal caudate to dorsal-ACC and AI in OCD	CSTC (DC) – SN ↑	ROI-based voxel-wise analysis

(Tomiyama et al. 2022b)	41 (16M, 25F; 33.34±11.7y)	49 (19M, 31F; 33.33±10.4y)	Increased FC between pre-SMA and IFG, BL-inferior parietal lobule, dACC and AI in OCD.	CSTC (SM) – FPN ↑<br>CSTC (SM) – DMN ↑<br>CSTC (SM) – SN ↑	ROI-based voxel-wise analysis
(Tomiyama et al. 2022a)	47 (18M, 29F; 33.3±11.9y)	62 (22M, 40F; 32.61±11y)	Increased FC between AI and PCC and within the CSTC (DC).	SN – DMN ↑<br>CSTC (DC) ↑	ROI-based voxel-wise analysis
(Vaghi et al. 2017)	44 (21M, 23F; 36.14±10.7y)	43 (22M, 21F; 37.51±12.1y)	Reduced FC between the caudate and vlPFC, and the putamen and DLPFC in OCD. Hyperconnectivity between basal ganglia and cerebellum	CSTC (SM) – CSTC (DC) ↓<br>CSTC (DC) – CSTC (limbic) ↓<br>CSTC (DC) – cerebellum ↑	ROI-based voxel-wise analysis
(Versace et al. 2019)	48 (18M, 30F; 23.3±4.5y)	45 (17M, 28F; 23.2±3.8y)	Decreased FC between ACC and mPFC in OCD.	SN – DMN ↓	ROI-based voxel-wise analysis
(Wang et al. 2019)	22 (11M, 11F; 22.41±6.2y)	22 (11M, 11F; 22.68±2.3y)	Increased FC between subregions of DMN and FPN. Decreased FC within DMN.	DMN – FPN ↑<br>DMN ↓	ROI-based voxel-wise analysis
(Weber et al. 2014)	11 (6M, 6F; 13±2.9y)	9 (5M, 4F; 12.7±3.2y)	Decreased FC between ACC, BL-DLPFC and increased FC within auditory network in OCD.	SN – CSTC (DC) ↓<br>DMN ↑	Parcellation-based network analysis
(Xia et al. 2020)	40 (22M, 18F; 22.48±6.1y)	42 (21M, 21F; 22.76±6.1y)	Increased FC within the SN (BL-AI and ACC) and BL-AI with the DMN	SN ↑<br>SN – DMN ↑	ROI-based voxel-wise analysis
(Xie et al. 2017)	68 (37M, 31F; 30.24±7.8y)	33 (17M, 16F; 25.61±7.4y)	Increased FC between dACC, caudate and AI. Decreased FC within FPN.	SN ↑ FPN ↓<br>SN – CSTC (DC) ↑	ROI-based voxel-wise analysis
(Xing et al. 2020)	61 (16M, 45F; 26.1±8.1y)	67 (23M, 44F; 21.3±5y)	Significantly lower FC within the cerebellum and between the cerebellum and inferior occipital cortex and thalamus in OCD.	Cerebellum ↓<br>Cerebellum – VN ↓	Parcellation-based network analysis
(Xu et al. 2019)	27 (16M, 11F; 29.22±8.1y)	21 (12M, 9F; 33.57±7.2y)	Decreased FC between cerebellum and several networks including DMN, affective-limbic and sensorimotor networks in OCD.	DMN – cerebellum ↓<br>CSTC (limbic) – cerebellum ↓<br>CSTC (SM) – cerebellum ↓	ROI-based voxel-wise analysis
(Xu et al. 2021)	36 (22M, 14F; 29.14±7.7y)	50 (27M, 23F; 30.2±7.2y)	Increased FC of caudate-OFC, ventral striatum (VS)-OFC, VS-mPFC, and putamen-SMA, and decreased FC of caudate-ACC, putamen-ACC, and putamen- DLPFC.	CSTC (VMC) ↑, CSTC (SM) ↑<br>CSTC (VMC) – CSTC (DC) ↑<br>CSTC (VMC) – CSTC (limbic) ↑<br>CSTC (DC) – SN ↓<br>CSTC (SM) – SN ↓<br>CSTC (SM) – FPN ↓	ROI-based voxel-wise analysis
(Xu et al. 2022)	40 (30M, 10F; 24.63±7.8y)	43 (18M, 25F; 24.16±4.3y)	Decreased FC between dmPFC-SMA and IFG-OFC in OCD.	CSTC (DC) – CSTC (SM) ↓<br>FPN – CSTC (VMC) ↓	ROI-based voxel-wise analysis
(Xu et al. 2023a)	100 (52M, 48F; 23.15±9.3y)	120 (57M, 63F; 22.17±5.9y)	Increased FC between R-AI to L-DLPFC, R-DLPFC to cerebellum, cerebellum to PCC and ACC. Decreased FC between L-AI to L-DLPFC, R-AI to ACC and within R-DLPFC.	SN – FPN ↑<br>FPN – Cerebellum ↑<br>Cerebellum – DMN ↑<br>SN ↓ FPN ↓	ROI-based voxel-wise analysis
(Xu and Zhang 2023)	103 (54M, 49F; 19±13y)	118 (56M, 62F; 22±8y)	Decreased FC within the cerebellum, CSTC-SM, DMN in OCD compared to HC.	Cerebellum ↓<br>CSTC (SM) ↓<br>DMN ↓	ROI-based voxel-wise analysis voxel-mirrored homotopic connectivity)
(Xu et al. 2023b)	73 (39M, 34F; 22.7±8.6y)	54 (30M, 24F; 22.28±6.8y)	Increased FC between angular gyrus and inferior parietal lobule (DMN), and between L-middle occipital gyrus (FPN) and cerebellum.	DMN ↑<br>FPN – Cerebellum ↑	ROI-based voxel-wise analysis

(Yang et al. 2019)	68 (45M, 23F; 27.99±8.2y)	68 (45M, 23F; 27.57±8.6y)	Significantly higher FC within the limbic CSTC and lower FC between putamen and SMA in OCD	CSTC (limbic) ↑<br>CSTC (SM) ↓	ROI-based voxel-wise analysis
(Ye et al. 2020)	73 (38M, 35F; 28.93±5.3y)	79 (42M, 37F; 27.73±5.7y)	Increased FC within the CSTC (SM) in PCG and decreased FC within SN (L-AI) in OCD.	CSTC (SM) ↑<br>SN ↓	ROI-based voxel-wise analysis
(Ye et al. 2021)	73 (38M, 35F; 28.93±5.3y)	79 (42M, 37F; 27.73±5.7y)	Decreased FC between the precuneus and vermis of the cerebellum in OCD	FPN – Cerebellum ↓	Parcellation-based network analysis
(Yu et al. 2022)	45 (26M, 19F; 28.19±7.9y)	45 (22M, 23F; 25.91±3.9y)	Increased FC between L-medial superior frontal gyrus and R-caudate in OCD.	FPN – CSTC (DC) ↑	ROI-based voxel-wise analysis
(Yun et al. 2017)	24 (17M, 7F; 24.9±6.7y)	34 (24M, 10F; 24±4.1y)	Significantly higher FC within R-ACC and L-DLPFC in OCD compared to HC	SN – FPN ↑	ROI-based voxel-wise analysis
(Zhang et al. 2011)	18 (14M, 4F; 23.3±5y)	16 (12M, 4F; 24.1±5.4y)	Increased FC between Mid-cingulate and PCC and within cerebellum. Decreased FC between AI and Post temporal cortex.	Cerebellum ↑<br>FPN – DMN ↑<br>SN – DMN ↓	ROI-based voxel-wise analysis
(Zhang et al. 2017)	23 (15M, 8F; 32.09±10.6y)	23 (15M, 8F; 31.39±10y)	Decreased FC of ACC-DLPFC and increased FC between dACC and caudate in OCD.	SN – FPN ↓<br>SN – CSTC (DC) ↑	ROI-based voxel-wise analysis
(Zhang et al. 2019)	30 (14M, 16F; 27.4±8.9y)	26 (10M, 16F; 27.8±10.2y)	Weakened FC between regions of CSTC and cerebellum (L-crus II, lobule VIII, R-striatum, cingulate) in OCD.	CSTC (SM) – Cerebellum ↓	ROI-based voxel-wise analysis
(Zhang et al. 2021)	58 (37M, 21F; 27.2±6.6y)	72 (34M, 38F; 24.4±3.4y)	Increased FC between L-caudate and BL-SMA in OCD.	CSTC (DC) – CSTC (SM) ↑	ROI-based voxel-wise analysis
(Zhao et al. 2021)	51 (32M, 19F; 27.25±7.5y)	25 (17M, 8F; 25.92±5.1y)	Decreased FC within the limbic CSTC loop and increased FC between SMA-putamen.	CSTC (limbic) ↓<br>CSTC (SM) ↑	ROI-based voxel-wise analysis
(Zhou et al. 2022)	85 (52M, 33F; 29.18±8.7y)	85 (51M, 34F; 28.16±10.9y)	Increased FC between BL-AI and BL-precuneus extending to SMA. Decreased FC between R-AI and lingual gyrus.	SN – FPN ↑<br>SN – CSTC (SM) ↑<br>SN – DMN ↓	ROI-based voxel-wise analysis

Table S4 - Resting-state fMRI studies including functional connectivity analysis method

Note. OCD – obsessive-compulsive disorder, fMRI – functional magnetic resonance imaging, M – male, F – female, y – years old, SD – standard deviation, NAc – nucleus accumbens, mPFC – medial prefrontal cortex, lPFC – lateral prefrontal cortex, CSTC – cortico-striato-thalamo-cortical, VMC – ventral motivational circuit, dACC – dorsal anterior cingulate cortex, l – left, r – right, BL – bilateral, DLPFC – dorsolateral prefrontal cortex, FPN – frontoparietal network, vmPFC – ventromedial prefrontal cortex, OFC – orbitofrontal cortex, SM – sensorimotor, DC – dorsal cognitive, OCPD – obsessive-compulsive personality disorder, PCC – posterior cingulate cortex, VN – visual network, SMA – supplementary motor area, AI – anterior insula, ROI – Region of Interest

Author (year)	OCD sample (Male, Female, Mean age, SD)	Comparison sample (Male, Female, Mean age, SD)	fMRI methodology	Key findings	Involved neurocircuitry and direction of connectivity findings	Functional Connectivity Analysis Method
(Admon et al. 2012)	13 (10M, 3F; 25.5±1y)	13 (10M, 3F; 27±0.5y)	fMRI collected during an interactive risky choice task	Reduced FC between amygdala-NAc and OFC-dACC in OCD.	CSTC (limbic) – CSTC (VMC) ↓<br>CSTC (VMC) – SN ↓	ROI-based voxel-wise analysis
(Agam et al. 2014)	21 (8M, 13F; 33±11y)	20 (11M, 9F; 33±11y)	fMRI collected during an anti-saccade task	Increased FC between the PCC and ACC in OCD.	DMN – SN ↑	ROI-based voxel-wise analysis
(Alves-Pinto et al. 2019)	39 (14M, 23F; 33.7±10.1y)	37 (16M, 23F; 32.2±8.3y)	fMRI collected during a reward learning task	Stronger effective and reward-related connectivity between vmPFC, L- and R-OFC, suggestive of dysfunction in parts of both DMN and CSTC networks.	DMN – CSTC (VMC) ↑	ROI-based voxel-wise analysis
(Cardoner et al. 2011)	21 (10M, 11F; 28.52±5.9y)	21 (10M, 11F; 26.2±3.4y)	fMRI collected during an emotional face-processing task	Increased FC between amygdala and DLPFC during task.	FPN – CSTC (limbic) ↑	ROI-based voxel-wise analysis
(Cocchi et al. 2012)	17 (8M, 9F; 32.8±10.8y)	19 (10M, 9F; 30.6±7.2y)	fMRI collected during a multisource inference task	Significantly higher FC within the SN (dACC-AI) in OCD.	SN ↑	Parcellation-based network analysis
(de Vries et al. 2014)	43 (22M, 21F; 38.1±9.7y)	37 (17M, 20F; 39.2±11.5y)	fMRI collected during a visuo-spatial n-back task	Increased FC between L-SMA and BL-amygdala, L-DLPFC and R-amygdala in OCD.	CSTC (SM) – CSTC (limbic) ↑<br>FPN – CSTC (limbic) ↑	ROI-based voxel-wise analysis
(Figee et al. 2013)	16 (9M, 7F; 45±9.7y)	13 (7M, 7F; 45±9.2y)	fMRI collected during a reward anticipation task	Hyperconnectivity in the frontostriatal networks in OCD is normalised with DBS to the NAc (particularly between NAc and mPFC and lPFC)	CSTC (VMC) – CSTC (limbic) ↑	ROI-based voxel-wise analysis
(Fitzgerald et al. 2010)	18 (6M, 12F; 13.9±2.6y)	18 (6M, 12F; 14.1±2.6y)	fMRI collected during a performance monitoring task and at rest.	Increased task-related dACC -vmPFC FC and decreased resting dACC-R operculum and vmPFC-PCC FC in OCD.	SN ↓<br>SN – CSTC (limbic) ↑<br>CSTC (limbic) – DMN ↓	ROI-based voxel-wise analysis
(Fontenelle et al. 2012)	11 (7M, 4F; 36.3±7.9y)	10 (6M, 4F; 34.8±7y)	fMRI collected during a sad mood induction task	Increased FC between ACC and ventral caudate and NAc in OCD	SN – FPN ↑<br>SN – CSTC (VMC) ↑	ROI-based voxel-wise analysis
(Hampshire et al. 2020)	20 (17M, 3F; 37.6±14.6y)	20 (15M, 5F; 36.3±8.3y)	fMRI collected during a stop signal task	Reduced FC between cerebellum-FPN and cerebellum-DMN in OCD	Cerebellum – FPN ↓<br>Cerebellum – DMN ↓	ROI-based voxel-wise analysis
(Han et al. 2016)	20 (12M, 8F; 25.5±5.4y)	21 (14M, 7F; 22.57±4.5y)	fMRI collected during a delayed-response working memory task	Emotional distraction significantly reduces FC between DLPFC-OFC in OCD compared to HC.	FPN – CSTC (VMC) ↓	ROI-based voxel-wise analysis
(Jaspers-Fayer et al. 2022)	23 (9M, 14F; 15.1±2.6y)	23 (7M, 16F; 14.2±3.1y)	fMRI collected during a Tower of London task	Increased FC between superior/middle frontal gyrus and precuneus/inferior parietal lobule.	FPN – DMN ↑	ROI-based voxel-wise analysis

(Jhung et al. 2014)	26 (20M, 6F; 27.25±6.1y)	18 (15M, 3F; 28.2±6.6y)	fMRI collected during a contamination provocation task	Contamination group showed significantly higher FC between ventral striatum (NAc) and R-insula.	CSTC (VMC) – SN ↑	ROI-based voxel-wise analysis
(Jung et al. 2013)	19 (12M, 7F; 25.84±7.15y)	18 (11M, 7F; 24.83±3.88y)	fMRI collected at rest and during a monetary incentive delay task	Resting state – increased FC between NAc and lateral OFC<br>Incentive processing – decreased FC between NAc and areas of the limbic CSTC (amygdala)	Rest: CSTC (VMC) ↑<br>Task: CSTC (VMC) – CSTC (limbic) ↓	ROI-based voxel-wise analysis
(Kim et al. 2020)	17 (12M, 5F; 26.4±6y)	21 (11M, 10F; 26±5.3y)	fMRI collected during a Tower of London task	At baseline, OCD group showed decreased FC between FPN and DMN.	FPN – DMN ↓	Parcellation-based network analysis
(Kim et al. 2022)	105 (70M, 35F; 25.05±6.6y)	99 (64M, 35F; 23.93±5.8y)	fMRI collected during a set shifting task.	Increased FC within the bilateral inferior-middle frontal gryus and between the anterior caudate-thalamus.	FPN ↑<br>CSTC (DC) ↑	Parcellation-based network analysis
(Koch et al. 2018)	44 (17M, 27F; 32.7±9.3y)	37 (15M, 22F; 32±8y)	fMRI collected during a monetary reward task	Increased connectivity between PCC and vmPFC (areas of the DMN) in OCD compared to HC.	DMN ↑	ROI-based voxel-wise analysis
(Lee et al. 2022)	41 (36M, 5F; 25.27±6.5y)	47 (46M, 1F; 22.59±1.9y)	fMRI collected during a thought-action fusion task	Decreased FC between Midcingulate cortex-AI, Middle temporal gyrus-amygdala, AI-precuneus in OCD	FPN – SN ↓<br>DMN – CSTC (limbic) ↓	ROI-based voxel-wise analysis
(Liu et al. 2023)	42 (25M, 17F; 21.86±4.9y)	48 (21M, 27F; 20.65±2.1y)	fMRI collected during a cued task switching paradigm	Increased FC within the FPN and between FPN and DMN in OCD.	FPN ↑<br>FPN – DMN ↑	ROI-based voxel-wise analysis
(Marsh et al. 2014)	22 (11M, 11F; 30±9.1y)	22 (11M, 11F; 30.14±9.4y)	fMRI collected during a Simon Spatial Incompatibility task	Increased FC between putamen and SFG, inferior parietal lobule and caudate in OCD.	CSTC (SM) – FPN ↑<br>CSTC (SM) – DMN ↑<br>CSTC (SM) – CSTC (DC) ↑	ROI-based voxel-wise analysis
(Paul et al. 2019)	21 (8M, 13F; 33.1±10.8y)	21 (8M, 13F; 33.1±10.1y)	fMRI collected during a symptom provocation task	Reduced FC between L-OFC and amygdala in OCD during symptom provocation.	CSTC (VMC) – CSTC (limbic) ↓	ROI-based voxel-wise analysis
(Picó-Pérez et al. 2022)	30 (13M, 17F; 28.97±11.14y)	29 (14M, 15F; 29.35±12.14y)	fMRI collected during a cognitive reappraisal task	Increased FPN connectivity (between left angular gyrus and left vlPFC) in OCD compared to HC during an emotion regulation task, and decreased FC in OCD when experiencing negative emotions.	FPN ↑	ROI-based voxel-wise analysis
(Ravindran et al. 2020)	31 (13M, 18F; 34±8.5y)	17 (9M, 8F; 32.6±9.2y)	fMRI collected during an emotion provocation task	Increased FC between PCC and visual cortices and CSTC regions. Checking subtypes – motor cortices, washing subtypes – anterior insula and OFC.	DMN – VN ↑<br>DMN – CSTC (limbic) ↑	ROI-based voxel-wise analysis
(Rus et al. 2017)	42 (15M, 27F; 32.5±10y)	37 (15M, 22F; 30.99±7.6y)	fMRI collected during a contamination provocation task	Increased FC between L-amygdala and parietal cortex in OCD.	CSTC (limbic) – DMN ↑	ROI-based voxel-wise analysis
(Schlösser et al. 2010)	21 (5M, 16F; 31.3±10.2y)	21 (5M, 16F; 28.8±8.3y)	fMRI collected during a modified Stroop task	Significantly higher FC between dACC and l-DLPFC in OCD.	DMN – FPN ↑	ROI-based voxel-wise analysis

(Stern et al. 2011)	39 (17M, 22F; 27.8±8.7y)	38 (18M, 20F; 28.9±9.1y)	fMRI collected during an incentive flanker task	Increased FC between vmPFC and AI, R-thalamus in OCD	CSTC (limbic) – SN ↑	ROI-based voxel-wise analysis
(Stern et al. 2017)	18 (7M, 11F; 28.2±7.1y)	18 (8M, 10F; 27.2±6.5y)	fMRI collected during internal/external attentional tasks.	Stronger FC between dmPFC and occipital regions in OCD	DMN – VN ↑	ROI-based voxel-wise analysis
(Thorsen et al. 2020)	31 (12M, 19F; 30.19±9.2y)	26 (8M, 18F; 31±10.7y)	fMRI collected during a stop signal task	Increased FC between R-amygdala and R-inferior frontal gyrus and pre-SMA.	CSTC (limbic) – FPN ↑<br>CSTC (limbic) – CSTC (SM) ↑	ROI-based voxel-wise analysis
(Vaghi et al. 2017)	21 (3M, 18F; 37.9±14.3y)	20 (5M, 15F; 36.45±8.5y)	fMRI collected during a Tower of London task	OCD group showed reduced FC between R-DLPFC and putamen.	FPN – CSTC (SM) ↓	ROI-based voxel-wise analysis
(van der Straten et al. 2020)	23 (10M, 13F; 33.48±2y)	23 (12M, 11F; 33.52±3.1y)	fMRI collected during a stress induction task	Stress induction caused significant reduction in FC between the caudate and precuneus in OCD.	CSTC (DC) – DMN ↓	ROI-based voxel-wise analysis
(van Velzen et al. 2015)	41 (21M, 20F; 38.6±9.8y)	37 (18M, 19F; 39.7±11.6y)	fMRI collected during a stop signal task	Decreased FC between inferior frontal gyrus and amygdala in OCD.	FPN – CSTC (limbic) ↓	ROI-based voxel-wise analysis

Table S5 - Task-related fMRI studies including functional connectivity analysis method

Note. OCD – obsessive-compulsive disorder, fMRI – functional magnetic resonance imaging, M – male, F – female, y – years old, SD – standard deviation, NAc – nucleus accumbens, mPFC – medial prefrontal cortex, lPFC – lateral prefrontal cortex, CSTC – cortico-striato-thalamo-cortical, VMC – ventral motivational circuit, dACC – dorsal anterior cingulate cortex, l – left, r – right, DLPFC – dorsolateral prefrontal cortex, FPN – frontoparietal network, vmPFC – ventromedial prefrontal cortex, OFC – orbitofrontal cortex, SM – sensorimotor, DC – dorsal cognitive, OCPD – obsessive-compulsive personality disorder, PCC – posterior cingulate cortex, VN – visual network, ROI – Region of Interest, ROI – Region of Interest
